# Inexpensive, non-invasive biomarkers predict Alzheimer transition using machine learning analysis of the Alzheimer’s Disease Neuroimaging (ADNI) database

**DOI:** 10.1371/journal.pone.0235663

**Published:** 2020-07-27

**Authors:** Juan Felipe Beltrán, Brandon Malik Wahba, Nicole Hose, Dennis Shasha, Richard P. Kline, For the Alzheimer’s Disease Neuroimaging Initiative

**Affiliations:** 1 Department of Computer Sciences, Courant Institute of Mathematical Sciences, New York University, New York, New York, United States of America; 2 Fordham University Lincoln Center Campus, New York, New York, United States of America; 3 The Center for Cognitive Neurology, Department of Anesthesiology, NYU Langone Medical Center, New York, New York, United States of America; Nathan S Kline Institute, UNITED STATES

## Abstract

The Alzheimer’s Disease Neuroimaging (ADNI) database is an expansive undertaking by government, academia, and industry to pool resources and data on subjects at various stage of symptomatic severity due to Alzheimer’s disease. As expected, magnetic resonance imaging is a major component of the project. Full brain images are obtained at every 6-month visit. A range of cognitive tests studying executive function and memory are employed less frequently. Two blood draws (baseline, 6 months) provide samples to measure concentrations of approximately 145 plasma biomarkers. In addition, other diagnostic measurements are performed including PET imaging, cerebral spinal fluid measurements of amyloid-beta and tau peptides, as well as genetic tests, demographics, and vital signs. ADNI data is available upon review of an application. There have been numerous reports of how various processes evolve during AD progression, including alterations in metabolic and neuroendocrine activity, cell survival, and cognitive behavior. Lacking an analytic model at the onset, we leveraged recent advances in machine learning, which allow us to deal with large, non-linear systems with many variables. Of particular note was examining how well binary predictions of future disease states could be learned from simple, non-invasive measurements like those dependent on blood samples. Such measurements make relatively little demands on the time and effort of medical staff or patient. We report findings with recall/precision/area under the receiver operator curve after application of CART, Random Forest, Gradient Boosting, and Support Vector Machines, Our results show (i) Random Forests and Gradient Boosting work very well with such data, (ii) Prediction quality when applied to relatively easily obtained measurements (Cognitive scores, Genetic Risk and plasma biomarkers) achieve results that are competitive with magnetic resonance techniques. This is by no means an exhaustive study, but instead an exploration of the plausibility of defining a series of relatively inexpensive, broad population based tests.

## Introduction

Alzheimer’s Disease (AD) is a neurodegenerative illness which affects the elderly, and whose public impact, therefore, will continue to grow as the population ages. In the final stages, patients lose their independence, as well as their cognitive abilities. Because early symptoms are similar in many respects to healthy aging, diagnosis can be delayed by mistaking cognitive change for aging [1]. So, the disease may progress for one or two decades without discernable symptoms but nevertheless, manifest classical neuropathological findings in the event of autopsy due to unrelated death. At the point where it is symptomatic and can be more readily diagnosed, it is extremely difficult to treat, since there is both a need to halt disease progression, as well as to undo prior neuronal damage.

In addition to aging, other factors play a role in the development and progression of the disease, including many of the same risk factors involved in diabetes, cardiovascular illness, stroke and metabolic syndrome. These risk factors include hypertension, hyperlipidemia, sleep disordered breathing, and systemic inflammation. Many genes and single nucleotide polymorphisms have been reported to accompany AD, but only the ApoE4 polymorphism has been extensively validated [2, 3]. Many of these risk factors cause reduced delivery of oxygen and substrate to the brain, with reduced removal of debris. AD is thus sometimes accompanied by reduced blood levels of metabolic regulators and nerve growth factors, which stimulate nerve growth and mitigate the impact of amyloid beta [4, 5].

The two main neuropathological findings in AD are amyloid-beta plaque deposits and hyper-phosphorylated tau peptides. Both are associated with a toxic neural environment [6], as well as increased rates of cortical atrophy and shape changes [7]. It is often unclear which factors are causal and which merely a result of the underlying pathology. Energy balance, of course, is also affected by mitochondrial dysfunction, which implicates an additional set of potential DNA mutations at disease onset [8].

Due to the variety of pathways potentially involved in AD progression, one could hypothesize their association with multiple biomarkers, especially those found in plasma. Those plasma biomarkers could supplement major prognosticators like cortical atrophy detected by MRI [9–11] or even stand alone. In addition, hypometabolism and impairment of amyloid clearance lead to alterations in well known PET and CSF biomarkers during various time frames of disease progression [12]. In this study, we explore the prediction of Alzheimer’s progression based on plasma biomarkers and non-linear machine learning techniques.

### Detectable alterations at an early stage

Early aspects of the disease include deterioration of cortical networks (e.g. the default mode network) as a result of lost neuronal viability and connectivity. According to the dissociation hypothesis [13], destruction of cortical networks involves both primary neural destruction, as well as atrophy of functionally related cortical regions, now deprived of input. Thus, primary atrophy would show up as reduction in volume on an MRI [14], whereas loss of input could show up as a reduced signal on FDG PET images [15, 16]–implying a hypo-metabolic neural state with reduced electrical activity. As cell survival in the network is reduced and with it network function compromised, a pattern of atrophy spreads through the cortex starting in the temporal lobes. This ultimately is accompanied by amyloid-beta deposits and hyper-phosphorylated tau protein [17]. Thus, the biomarker changes we are looking for stem from both primary effects due to disease pathology as well as secondary effects due to disease related neural dysfunction.

### Monitoring and interpretation of large numbers of biomarkers

Whatever is driving the abnormal neurochemistry or neurophysiology associated with AD is also causing alterations in the levels of intermediaries that populate the underlying pathways. There are many scenarios potentially associated with the course of AD, so we are inclined to cast a wide net to look for these alterations. The ADNI database has values for 145 plasma biomarkers (BM), as well as over 300 anatomical measurements from MRI regions of interest (ROI). In terms of plasma biomarkers, some of these are byproducts of inflammation (inflammatory cytokines and stress hormones), related to neural plasticity growth factors–e.g., brain-derived neurotrophic factors (BDNF), or metabolic activity regulators like leptin or insulin. Although changes in these pathways normally might be benign and reversible, when stressed and near the threshold of stable function, these changes could have serious clinical consequence. Modeling the complex relationship between such a large number of biomarkers, with few strong priors on their relationship or distribution lends itself to modern machine learning approaches.

Well known models (Jack, Knopman et al. 2013) have presented time plots showing the onset of symptoms and BM intensities as a function of time of progression. This suggests that a multimodal model can take values of certain BMs, even those with no direct relationship, and use them to place the subject within a progression time frame, or in reference to a time anchor. In this study, we focus on predicting transition from mild cognitive impairment (MCI) to full Alzheimer’s disease (AD) using non-linear machine learning techniques. This transition is a well populated clinical milestone in the ADNI database and serves as a useful subset for exploratory studies on disease progression. Non-linear machine learning methods support such predictions without requiring the data fit a particular kind of smooth function.

In addition, due to the significant interest in events occurring at the earliest detectable times, prior to standard diagnostic reach, we also look at the potential to distinguish between NL and early MCI (stable MCI, see below). It is difficult to enroll NL patients who transition to MCI during their participation in the ADNI study, since they are scarce at baseline, and it is unclear when or if they will contract the illness. Thus we examine the period of disease inception by comparing biomarker values between the two cohorts (NL and stable MCI).

### Machine learning approaches

Classification and regression trees (CART) are a type of supervised machine learning algorithm, where supervised refers to the use of labeled data to build a model in order to make predictions on unlabeled data. In our initial approach, the label indicates a prediction as to whether a patient will transition from mild (MCI; mild cognitive impairment) to severe Alzheimer’s (AD). We then subsequently used the label to classify patients as normal or baseline early MCI. This was done based on our hypothesis that biomarkers values were again different between the two labelled groups

The main advantage of CART is its intuitive interpretability, as the trained model is a dichotomous tree, on which a researcher could trace the classification of any sample by hand. There is no statistical requirement as to the distribution of the input predictors, e.g. normality; nor is the quality of predictions compromised by the addition of redundant, otherwise colinear or useless variables. One limitation of CART is its greediness. When building each split on the labeled data the CART algorithm tries to maximize the “purity” of the split at that step. This greediness can easily lead to locally-maximal performance while missing a global optimum.;

A more important limitation of CART is that it cuts the solution space into hypercubes (e.g. if a patient has marker X value below 5 and marker Y value above 7, it defines a quadrant in a two dimensional Cartesian coordinate system). This can be undesirable if the relationship is more complex (e.g. the product of X and Y should be below 25). One way to combat both such limitations is to use many trees, yielding a Random Forest (RF) classifier; where many short decision trees are generated with a randomly determined feature at each split, and their decisions averaged. A closely related approach is called Gradient Boosting (GB), where regression trees are fit in series, each trying to fit (or fix) the error of the previous weak learner. We compare CART, Random Forests and Gradient Boosting in this paper and find that the latter two give about the same performance and both out-perform CART. Support vector machine (SVM) approaches, also tested, are known to perform best on small sets of predictor variables and show their utility here on pre-compressed sets of variables.

## Methods

The participants in the ADNI study provided written consent. The anonymized data was then distributed by ADNI to approved investigators with ADNI projects. The use of this subsequently de-identified data was reviewed but not considered human subject research by my institutional review board (NYU Langone Medical Center).

Our experiment has 320 patients with 525 different features available. However, some approaches select features with high importance and perform the analysis just on them. We also tested compression strategies using a 2-dimensional cognitive performance feature provided in ADNI, and a PCA derived 5 feature basis set from the baseline MRI data. The classification task is challenging in that it has a bigger feature-space than the number of samples and very few features are present for every single patient. In order to classify the patients efficiently while avoiding overfitting, we dropped features with missing data, and we use an ensemble classification algorithm to avoid the challenges of classification in high- dimensional spaces (referred to as the curse of dimensionality) [18].

For every classification task with standard category and regression tree (CART) analysis, we perform leave-two-out cross-validation. We train the model on all data points but two, and then predict the group label for the left out data points. Each data-point have been left out once, we measure the performance of the model by looking at the predictions of all left out data points. Additionally, we set aside a test set of 45 patients that will never be seen during training for more stringent evaluation. As mentioned above, first, we try classifying these two groups using a standard Classification And Regression Tree (CART) [19]. In our case, we used the Gini Impurity splitting criterion, the default choice in Python's scikit-learn library.

Also as mentioned above, while in standard CART, each node is split using all available variables, in Random Forests (RF; [20]), each node is split among the best of a subset of predictors randomly chosen at that node. Thus, there are two parameters that have to be set for RF–the number of trees (n_tree_) in the forest, and the number of variables (m_try_) considered in the subset at each node. The final prediction comes from the aggregation of the majority vote of the n_tree_ trees. Results below use the following values for these two parameters, n_tree_ = 200, and m_try_ = 5. (See S1 Appendix for schematic illustrating the difference between CART and RF.)

Two nice properties of RF are that (i) random forests approaches model complex non-linear functions that may not be (and in general are not) expressible in closed form; (ii) as for CART, the addition of variables, highly correlated with current variables or completely irrelevant, does not degrade the importance of the original set of variables. We also used several analyses with the gradient boost technique, comparing results for the best set of features (by AUC value) used with RF. We also employed Support Vector Machines (SVM) with a radial basis function kernel. As suggested in the literature, SVMs do well generating predictions from small sets of input variables–for example those using data compressed with Principal Component Analysis (PCA). With the exception of RandomForest’s number of trees, all hyperparameters were set to their default scikit-learn values.

In order to compare the performance of all of these predictors, we calculated the area under the receiver operating characteristic (ROC) curve and the area under the precision-recall curve. The former is less sensitive to class imbalance while the latter provides the more interpretable result. We analyzed both of them in this study but used ROC to determine which classifier and feature combinations were most effective.

### Data used in this study

*Data used in the preparation of this article were obtained from the Alzheimer’s Disease Neuroimaging Initiative (ADNI) database (adni*.*loni*.*usc*.*edu)*. *The ADNI was launched in 2003 as a public-private partnership*, *led by Principal Investigator Michael W*. *Weiner*, *MD*. *The primary goal of ADNI has been to test whether serial magnetic resonance imaging (MRI)*, *positron emission tomography (PET)*, *other biological markers*, *and clinical and neuropsychological assessment can be combined to measure the progression of mild cognitive impairment (MCI) and early Alzheimer’s disease (AD)*. *For up-to-date information*, *see* *www*.*adni-info*.*org*.

In the ADNI I database, there were 1435 research identification numbers (RID; each uniquely assigned to a subject) for which n = 815 were populated by demographic data including age, gender, BMI, ApoE4 status, systolic blood pressure and years of education. We focused on ADNI I, since ADNI II and “GO” did not yet report plasma BMs. (Later populations did have CSF amyloid beta and hyperphosphorylated tau values, but not the 145 plasma biomarkers.) The data used in this study was downloaded on Jan 30, 2014. More recent data will be reserved for a subsequent study looking at slightly different feature comparisons. There were 566 subjects with plasma analyte BMs. Most individual BMs sampled occurred for all subjects.

MRI values were well populated in ADNI I with ~815 subjects having MRI region of interest (ROI). Variables were acquired independently from each hemisphere with various dimensions including volume, thickness, and/or cortical area. For subjects diagnosed as MCI at baseline (i.e., our target population), there were 327 total MRI variables and 341 subjects. This representation of MRI-based anatomy, using raw data for all 815 ROIs, in whatever dimensions they were listed, was called MRI1. No attempt was made to extrapolate to volume. A second baseline MRI feature set (MRI1norm) was derived from 18 volume based ROIs, normalized to subject intracranial volume. This reduced the variance due to subject cranial size.

Annual rates of atrophy is a very important predictor of progression [21, 22]) This was calculated as annual percent change (‘APC’) for a small subset (n = 19) of important ROIs using a linear estimate. We calculated the difference between baseline and 6 month volumes, divided by baseline value, multiplied by 100 to attain percent change. This value was then doubled to annualize the atrophy rate. Values were calculated separately for each hemisphere as the feature set called MRI2; and derived from volume based measurements from hippocampus, entorhinal cortex, precuneous, posterior cingulate, amygdala, lateral ventricle, middle temporal lobe, total white matter cortex, and total gray matter cortex.

A final MRI representation, the third one derived solely from baseline values, was a compressed set of MRI variables (MRIPCA). This composite of many MRI variables at baseline, was derived using principal component analysis (PCA) producing a 5 eigenvector (EV) basis set from 42 ROI ADNI variables. These 42 ROI variables were determined by Risacher [23, 24] to be the best predictors of conversion from MCI to AD. We confirmed that at least two components of this set of EVs (EV#1 and EV#3) significantly correlated with diagnosis (NL, MCI, AD; [25]), and also with executive function (EV#1) and memory (EV#3) We used the PCA subroutine from SPSS version 24, selecting EVs with coefficients > 1.0 in the Spree plots. ROIs for EV#1 and EV#3 (coefficients > 0.4) follow. The most prominent vector (EV#1) represents many temporal lobe and other regions including: left (L) and right (R) precuneous cortical thickness (CT); L & R fusiform gyrus CT; L & R inferior (INF) parietal CT; L & R INF temporal CT; L & R middle temporal CT; supramarginal gyrus CT; L & R superior temporal CT. The second most prominent vector (EV#3) includes: L & R inferior lateral (lat) ventricle (vent), L & R lat vent, and CT of R entorhinal cortex. Thus 4 feature sets expressed the MRI acquired data, 3 based only on baseline data. The number of variables in each varied considerably, as did the most appropriate machine learning method to apply.

### Composite cognitive test scores

ADNI investigators have derived and posted composite scores for executive function (ADNI-EF) and memory (ADNI-MEM) using data from the ADNI neuropsychological battery and applying modern psychometric methods. ADNI-EF and ADNI-MEM have been described by the ADNI investigators [26, 27], and their values are currently available in the database.

There were many cognitive tests administered to subjects at different intervals. The composite memory score (ADNI-MEM) included: Rey Auditory Verbal Learning Test (RAVLT), ADAS Cog, Logical Memory and Mini-mental state exam (MMSE). The latter (ADNI-EF) includes values from: Category Fluency—Vegetables, WAIS-R Digit Symbol Substitution, Digit Span Backwards, Trails A and B, and 5 Clock Drawing items (circle, symbol, numbers, hands, time). These scores were available from 815 subjects in ADNI I.

Since they represent a novel variable, we present descriptive statistics here, namely (mean ±STD error, min, max): for ADNI Mem at baseline, (0.0304 + 0.030, -2.266, 2.599) and ADNI EF at baseline (-0.0425 ± 0.0337, -2.7600, 2.3620).

Some biological predictors (PET imaging, Amyloid beta, hyper-phosphorylated tau), although indicated as important diagnostics in the literature were not used in this analysis; since the measurements were not available for enough of the subjects at different ADNI enrollment sites, and also didn't overlap (by subject) with other critical variables like MRI and plasma biomarkers.

### Data sets considered in this study

In order to explore how examining multiple modalities worked in practice, we employed a variety of scenarios using different combinations of the following feature groups: (i) baseline plasma biomarkers; (ii) ADNI Memory and Executive Function composite scores at baseline; (iii) demographics, including gender, BMI, age and systolic blood pressure as well as ApoE4 related risk; (iv) over 320 MRI regions of interest (MRI1); (v) 5 principal component EVs derived from a select group of 42 MRI ROI (MRIPCA); and (vi) atrophy rates of 19 specific ROIs chosen for their relevance for predicting AD progression from the literature (MRI2), and (vii) 18 values from baseline MRI normalized to intracranial volume (MRInorm). We looked at these cohorts alone and in different combinations. Subgroups of the plasma biomarkers were chosen based on the literature (PlasmaAlzRelevant), including our own published work ([25, 28] also see Discussion.); but were also explicitly identified from the “kits” marketed by Myriad Rules Based Medicine, the company that processed the multiplexed immunoassays used by ADNI. These kit categories included the following (with number of assays in parenthesis): cardiovascular (n = 77), inflammation (n = 46), neuro (n = 35), and metabolic (n = 10). We also examined the entire plasma set as a group.

### Up-to intervals

Predicting binary outcomes (stable, progressive) for the transition from MCI to AD is complicated by the censoring effect of people leaving the study before reaching an end point, or the data acquisition being temporarily closed for data release. Both could occur before an individual reached a transition point. Therefore, we define “up-to intervals” where transitions are status changes within the time interval relevant to the patient. An up-to interval is defined by the end time point (t_f_). Patients qualify for analysis if they are either still MCI (Mild Cognitive Impairment) when t = t_f_ (i.e. “stable”), or they have progressed to AD for t < = t_f_ (“transitioned”).

**Fig 1:**
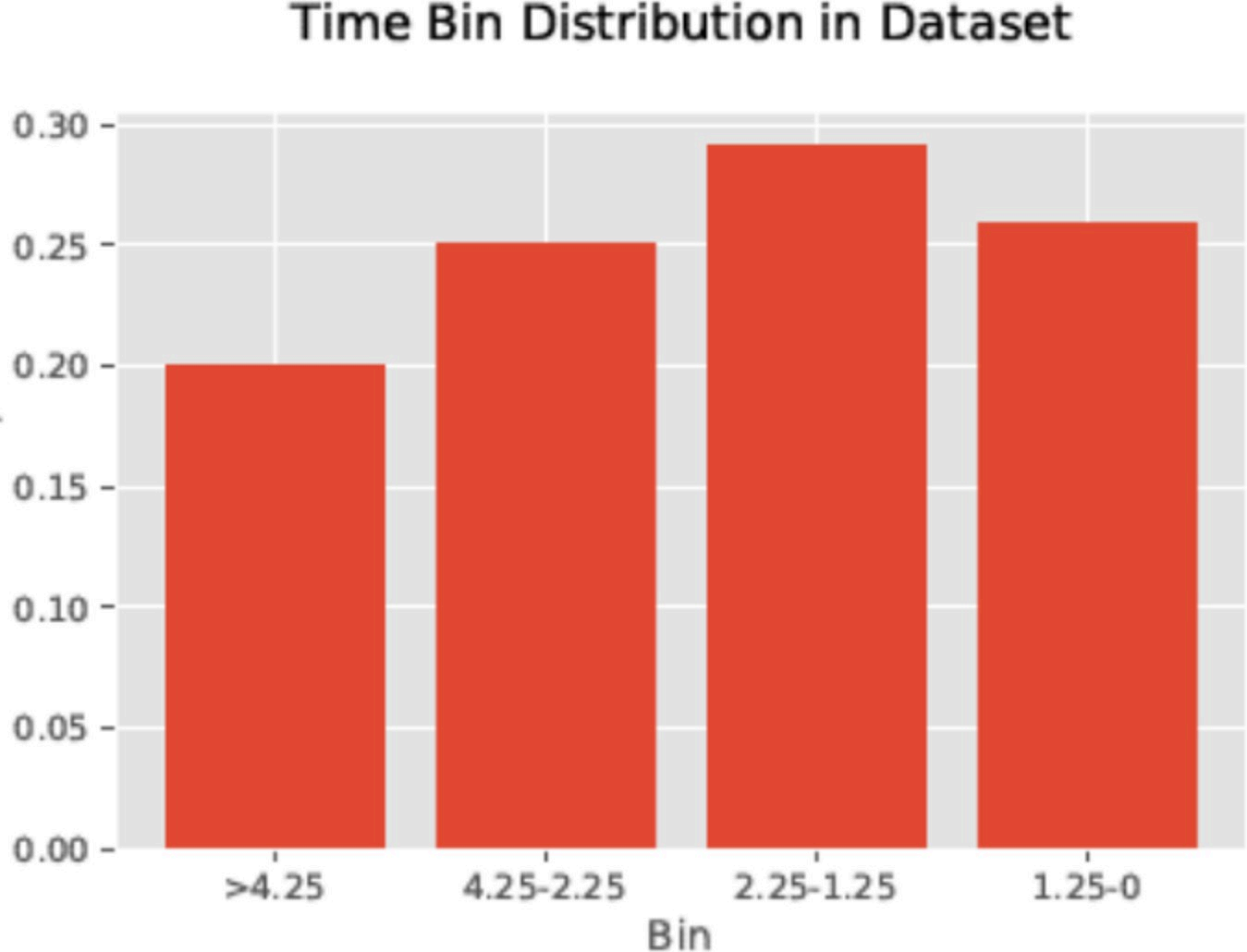
Distribution of subject assessment by time bins. Shows the distribution of elapsed times (by bins) at the point of subject transitioning, having data exported, or having main binary determined. The bar graph displays the proportion of subjects whose status was determined during 4 specific time intervals: prior to 1.25 yrs, 1.25 to 2.25 yrs, 2.25 to 4.25 yrs, and beyond 4.25 yrs. Note that target value ‘1’ subjects included those who transitioned after 2.25 years, or were declared stable at least once at or after that same point.

Due to the discrete time points at which particular cognitive tests scores or BM measurements were administered (acquired)–points at which patients medically transition or administratively exit from the study—tended to cluster. We divided clusters at the troughs between them and defined the up-to intervals considering these troughs. First, patients were assigned target variables by their status at a certain time point. For the *t* = 2.25 year target time frame, used in the results reported below, patients who remained in the study at 2.25 years from baseline and were still considered MCI were assigned a target value of ‘1’ (“stable”). Patients in severe or advanced Alzheimer’s (AD) before 2.25 years were assigned a value of ‘0’ (“transitional”), regardless of whether they remained in the study after the transition. This particular time frame (baseline to 2.25 years) was chosen in order to achieve a balance in the two patient outcomes (Fig 1). For the analysis of earliest changes, we compare any combination of feature values between normal subjects at baseline, a subjects identified as stable in the up-to analysis, i.e. subjects diagnosed as MCI at baseline and stable for 2.25 years. Rather than predicting transitions of individual subjects, we compare feature value averages between these two cohorts of subjects. The comparison is done using Random Forests, shown to be the most effective ML technique in the prior analysis.

## Results

In this section, we compare mean AUC measurements averaged over 20 random test subject groupings to determine how the 4 machine learning approaches (CART, GB, RF, & SVMs) compare. (Each bar of a mean value also has an error bar based on the standard error.) We further examined which feature groups are most successful in this same analysis at predicting the transition from incipient MCI to full Alzheimer’s Disease.

Each ML approach was applied to different combinations of features including four groupings of MRI (3 baseline representations: MRI1, MRI1PCA, MRI1norm) plus cortical atrophy rates (MRI2); five groupings of plasma biomarkers (RBM kits: “cardiac”, “inflammatory”, “metabolic”, “neuro”; a selection by authors based on the literature, “plasmaAlzRelevant”), plus demographics (BMI, Systolic BP, Age, gender), genetic risk (ApoE4) and compressed cognitive scores provided by ADNI (memory, executive function). To avoid the enormous number of permutations required to look at all combinations of features, we first examined individual feature groups as well as pairs of groups, where we required at least one prediction better than random per group; i.e. at least one classifier with a median performance above 0.5 AUC allowed us to examine the feature group. We selected only one feature group from each modality at a time (with the exception of pairing atrophy with baseline MRI).

For groups with many features, Random Forest and Gradient Boost gave the highest AUC scores, with RF values greater than GB for more than 90% of the feature inputs. The next two figures display AUC means over the 20 subject cohorts using bar graphs. We classified predictions where the mean AUC > 0.7 as ‘useful’, as well as where AUC > 0.75 as ‘good’. ‘Significant’ were those with the entire 95% AUC error bar > 0.7. There were 6 runs with ‘significant’ performance, and another 6 with ‘useful’ or ‘good’ predictions from over 100 analyses (or feature groupings).

**Fig 2:**
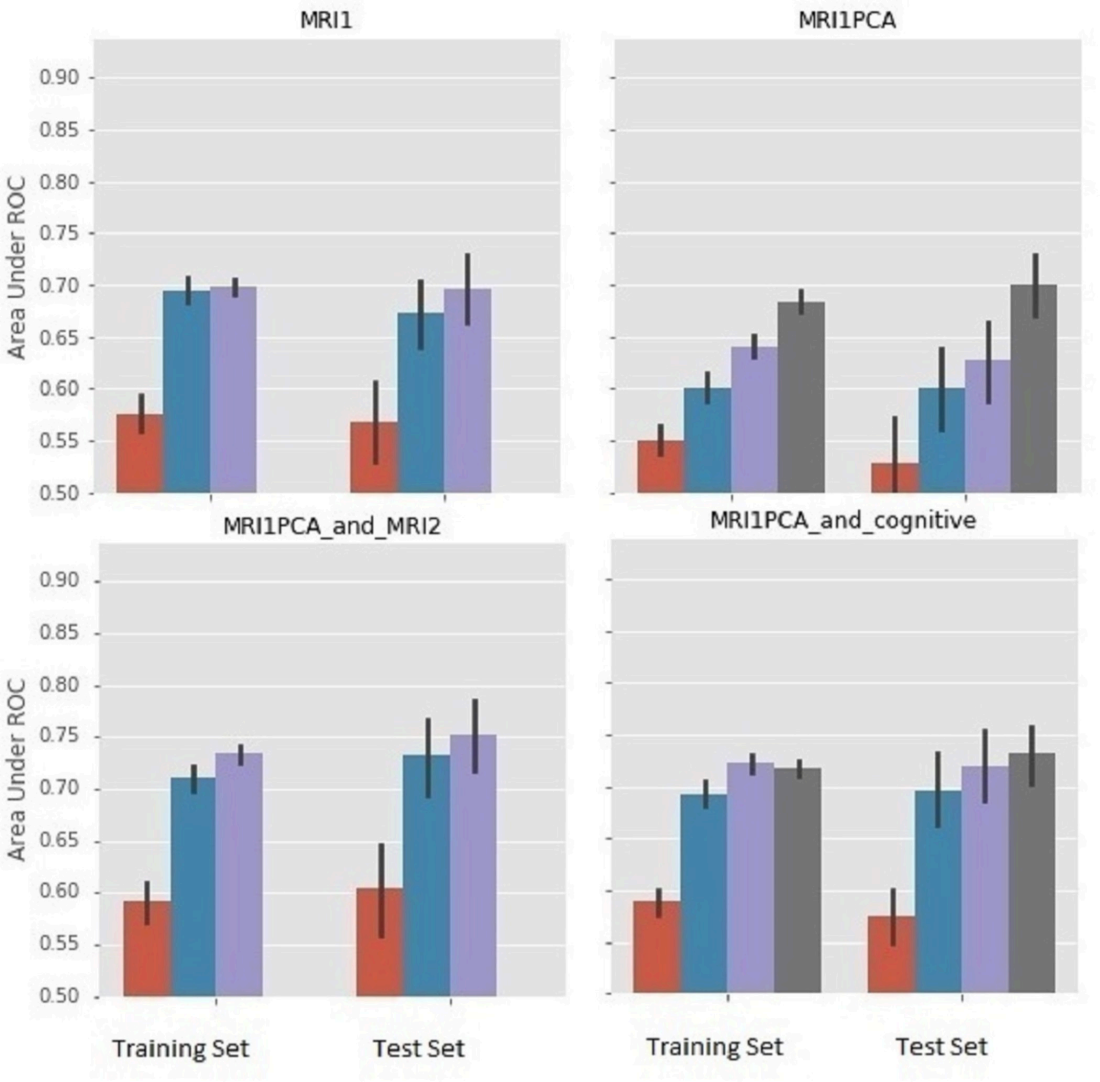
Comparison of different MRI representations & combining baseline MRI data with cognitive or MRI atrophy data. Color coding for bars: red–CART, blue–gradient boost, GB; purple—random forest, RF; dark gray–support vector machine, SVM. For each plot, bars on the left represent performance using leave-two-out cross-validation on the training set, whereas bars on the right represent performance using an independent 45 person test set. The test set was never seen in training and was randomly selected in that it was the complement to the randomly selected training set. We interpreted results from the test set.

We derived one figure (Fig 2) focused on MRI panels to compare the results from two different MRI representations (MRI1 and MRI1PCA; top panels), as well as the impact of adding other modalities (cognitive features and atrophy rates; bottom panels).

In Fig 2, the feature grouping with fewer variables (MRI1 PCA) leads to much larger AUC values for SVM (AUC > 0.7). For MRI1, with many variables, SVM is approximately 0.5, whereas RF is nearly 0.7. Addition of cognitive or atrophy data (MRI2) improves AUC values for MRI1 PCA. The entire SVM error bar of the AUC mean (derived from the 20 subject cohorts) is > 0.7 for MRI1PCA plus Cognitive, and the entire RF error bar is >0.7 for MRI1 PCA plus MRI2. The MRI values normalized to cranial volume (MRI NORM) gave at best an AUC of only 0.65 (RF), examined alone. Combining MRI1norm with atrophy (MRI2) resulted in mean AUC for RF of 0.74. Combining MRI1norm with cognitive produced a result with nearly equal predictive value (AUC = 0.72) for 3 of the ML approaches: RF, GB and SVM (not shown). Thus different MRI representations appear to have different utility depending on which other features they are combined with.

**Fig 3:**
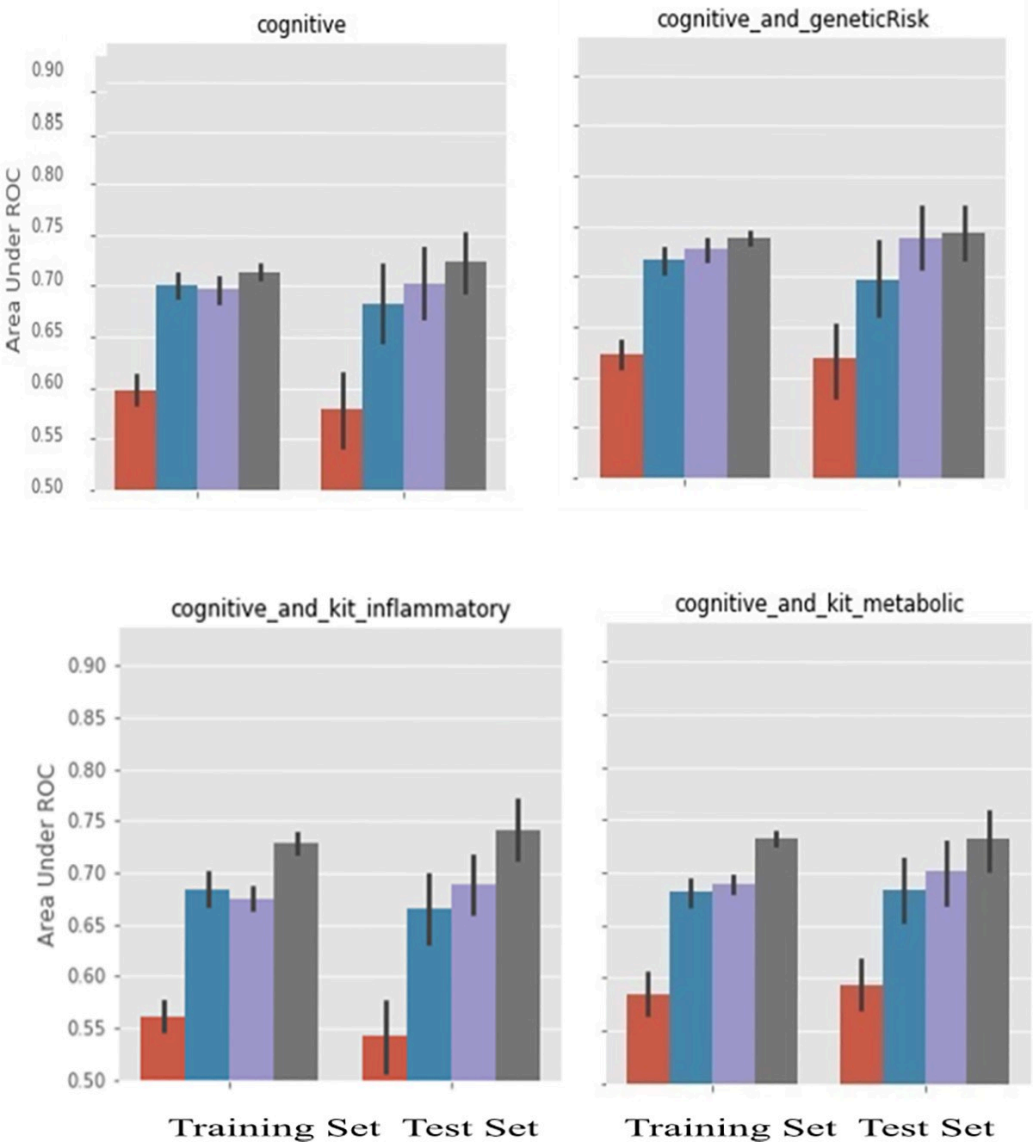
Further comparison of AUC for feature groups focused on non-invasive, easily acquired modalities. As in Fig 2, for all panels, left bars were training AUC results and right bars were test AUC results, the latter being used in our interpretations. The 4 bars show mean AUC as a function of ML technique (left to right, CART, GB, RF, and SVM; same color scheme as Fig 2). Cognitive features used in all panels. Top right also includes genetic risk. Bottom two panels also include plasma biomarker kits (inflammatory (L), metabolic (R)).

A second similar figure (Fig 3) compared several non-invasive, relatively inexpensive and easily acquired feature groupings, which excluded imaging. Thus, cognitive was shown with or without the genetic risk variable (upper right panel), and with one of two plasma biomarker groups each (kit inflammatory, or kit metabolic; bottom left and right respectively) for the other two panels.

In summary, 1) MRI PCA and cognitive were the only single modality tests with mean AUC >0.7 for at least one ML approach; 2) MRI Norm plus Cog was the only test result where all of RF, GB and SVM had mean AUC values > 0.72; 3) Cog plus any of the following—MRI2, MRI PCA, Genetic Risk, or pBM kit inflammatory; and MRI PCA plus MRI2 all had the entire AUC error bar above 0.7. So among the pairs and singlets of modalities, those including Cog, Genetic Risk and pBM kit inflammatory did very well, as did those containing both static and atrophy related MRI measurements. MRI PCA also had among the highest AUC values, when looking at the SVM measurement, since it was the MRI data representation with by far the smallest number of variables. CART itself, with no built-in protection against over definition, was consistently the least predictive of the 4, and, with few exceptions, showed smaller AUC values than the other ML techniques. (This point is demonstrated by plotting analysis results with ROCs in the S2 Appendix; as mentioned earlier, a diagrammatic aide is in the S1 Appendix). Of the above results, the one that was most surprising and most promising from a public health perspective was that Random Forests or Gradient Boosting applied to relatively easily obtained measurements (Cognitive scores, Genetic Risk and plasma biomarkers) achieve results that are competitive with magnetic resonance techniques.

### Random forest classifier on small feature set including plasma BMs plus cognitive

**Fig 4:**
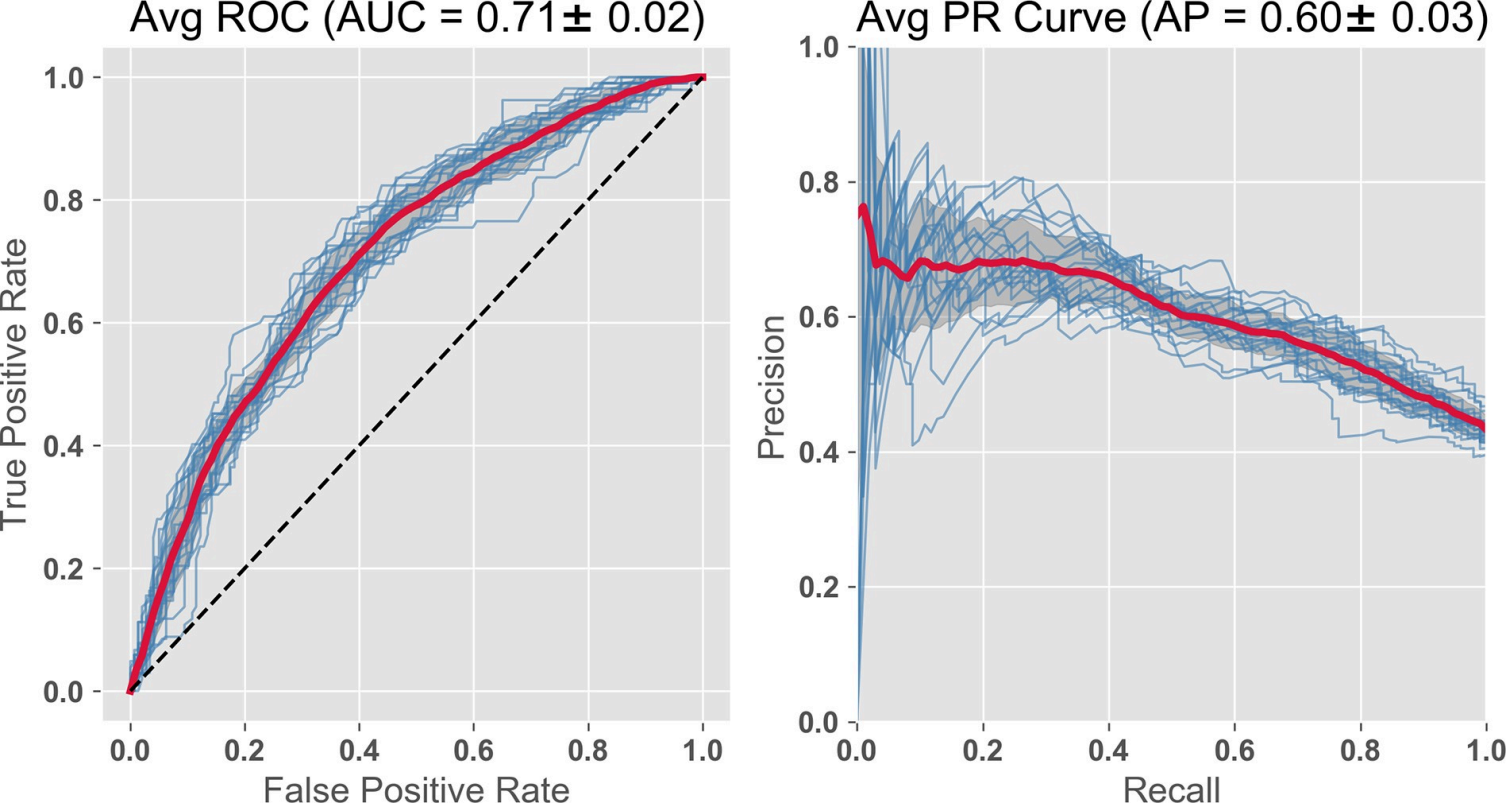
RF Classifier with subset of plasma BMs, compressed cognitive scores, and demographics. The features include the 2 compressed ADNI cognitive variables, 14 plasma analytes chosen from the literature (including 9 Apolipoproteins (Ai, Aii, Aiv, B, Ci, Ciii, D, E, H) plus leptin, insulin, CRP, vitronectin). Demographics included, systolic BP, BMI, age, gender. The importance is displayed as the GINI coefficient below in Table 1. AUC = 0.71; PR curve = 0.60.

In this study we are particularly interested in plasma biomarkers due to the low cost of acquisition and analysis, and ease of obtaining blood samples during normal clinical visits. Using a feature set consisting only of a subset of plasma BMs plus the cognitive and demographic baseline variables, the prediction has an ROC AUC = 0.71 (Fig 4).

The relative importance of the features, as determined from the GINI coefficients, are shown in Table 1. This exercise highlights the importance of the cognitive features followed by several of the apolipoproteins and BMI. Leptin, insulin, C Reactive protein and vitronectin have been identified as important in other studies.

**Table 1:** GINI Importance of features including plasma biomarkers.

Feature	Coefficient	Feature	Coefficient
ADNI_Executive Function	0.1383	Apolipoprotein AI (mg/mL)	0.0438
ADNI_Memory	0.1000	Apolipoprotein AIV (ug/ml)	0.0417
Apolipoprotein D (ug/ml)	0.0571	Apolipoprotein H (ug/mL)	0.0416
BMI	0.0550	VSBPSYS	0.0410
Testosterone (ng/ml)	0.0520	Leptin (ng/mL)	0.0413
Apolipoprotein Ci (ng/mL)	0.0500	Age (yrs)	0.0394
Apolipoprotein B (ug/ml)	0.0499	Insulin (U/mL)	0.0378
Apolipoprotein Aii (ug/mL)	0.0467	C Reactive Protein (ug/mL)	0.3733
ApolipoproteinCiii (ng/ml)	0.0460	Gender	0.3383
Vitronectin	0.0439	Apolipoprotein E (ug/ml)	0.00439

### Random forest classifier substituting MRI eigenvectors for plasma biomarkers, and combining both with atrophy features

**Fig 5:**
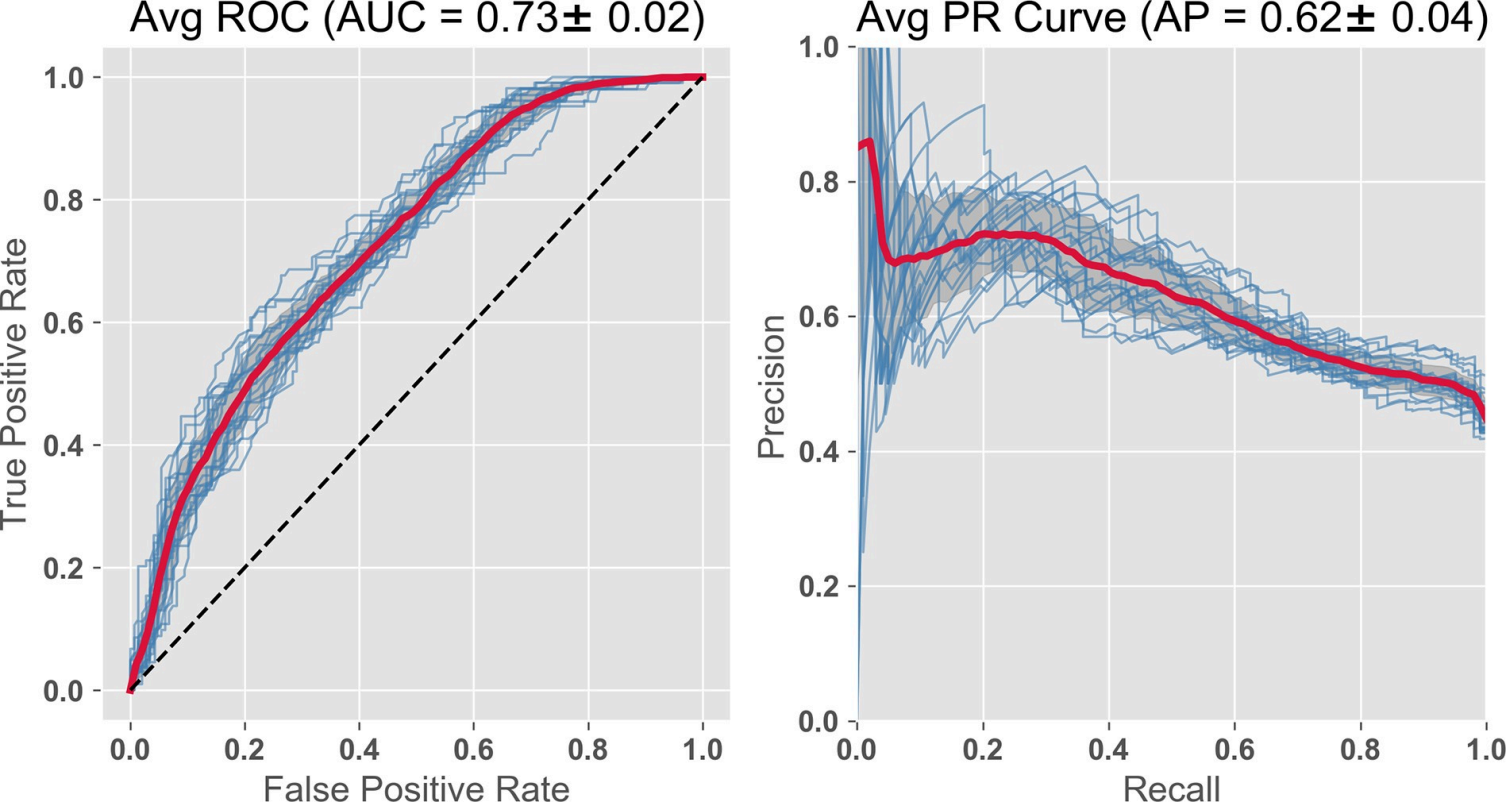
RF classifier with compressed cognitive scores, baseline MRI, and demographics. The features include the 2 compressed ADNI cognitive variables, the 5 Eigenvectors from PCA compression of MRI, and demographics. AUC = 0.73; PR curve = 0.62, both slightly improved by the substitution.

In this subsequent run, we compared the efficacy of plasma BM and MRI, the latter a diagnostic standard. We substituted the MRI PCA group for the 14 pBMs. This PCA representation is a feature set more comparable in numbers to pBM, since the MRI1 feature groups is several hundred variables. We have also shown that two of the eigenvectors in this group correlate well with the two ADNI compressed cognitive variables (ADNI EF, ADNI Mem) giving some functional interpretation to these eigenvector features [25]. Both feature groups had the two ADNI cognitive variables, plus the demographic/vital signs age, BMI, systolic blood pressure, ApoE4 risk and gender. As with pBMs, the predictions based on MRI were improved considerably by the addition of the two cognitive composite variables, increasing AUC from AUC = 0.65, to AUC = 0.73 (Fig 5).

**Fig 6:**
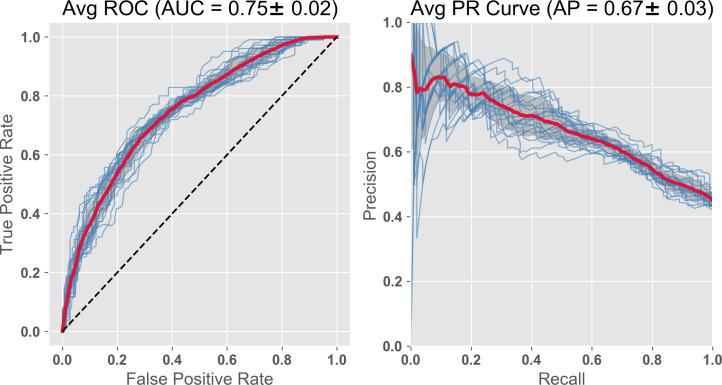
RF Classifier with subset of plasma BMs, compressed cognitive scores and MRI, and demographics. The features include the 2 compressed ADNI cognitive and MRI variables, 14 plasma analytes chosen from the literature and demographics. AUC = 0.75, PR curve = 0.67. This analysis includes all the features used in both Figs 4 and 5 resulting in a slight improvement for both analysis parameters.

Of interest is whether we can continue to improve predictions by adding important groups of these features together. Combining the plasma based and MRI based feature sets from the last two analyses gives us an improvement in the prediction (AUC = 0.75; Fig 6.; Table 2).

**Table 2:** GINI importance of features from MRI eigenvectors and plasma BMs.

Feature	Coefficient	Feature	Coefficient
ADNI_Compressed Executive Function	0.1153	Apolipoprotein H (ugmL)	0.03112
Apolipoprotein E (ug/ml)	0.0855	VSBPSYS (systolic BP)	0.03087
ADNI_Compressed Memory	0.0801	Apolipoprotein Aiv (ug/ml)	0.03077
CReactiveProtein (ug/mL)	0.0666	Insulin (U/mL)	0.03057
Apolipoprotein D (ug/ml)	0.0453	Age (yrs)	0.02962
BMI	0.0440	Leptin (ng/mL)	0.02950
MRI Eigen Vector #4	0.0380	Vitronectin (ug/ml)	0.02869
Apolipoprotein B (ug/ml)	0.0362	MRI Eigen Vector #3	0.02839
Apolipoprotein Ci (ng/ml)	0.0352	MRI Eigen Vector #1	0.02728
Apolipoprotein Aii (ng/ml)	0.0346	MRI Eigen Vector #2	0.02575
Apolipoprotein Ciii (ug/ml)	0.0341	Gender	0.02426
Apolipoprotein Ai (mg/ml)	0.0333	Testosterone (ng/ml)	0.002845
MRI Eigen Vector #5	0.0324		

lists in order of decreasing coefficient size the features in order of the diminishing of their importance. Among the top 7 most important features are the composite executive function (EF) score and composite memory (Mem), an MRI eigenvector, 3 plasma biomarkers and a demographic. EV#4 includes: L. accumbens vol, R. ventral dorsal column vol, L. & R. amygdala vol, L. & R. hippocampal vol, R. entorhinal vol.

In short, different sets of features combine to represent different dynamics, causing particular features to climb or fall in significance due to collinearities, other relationships, or proximity to clinical milestones. A set of features derived from small blood samples would be ideal for mass screenings and tracking studies. Optimization by combining multiple modalities could provide a more precise diagnostic for clinics having greater resources and time.

**Fig 7:**
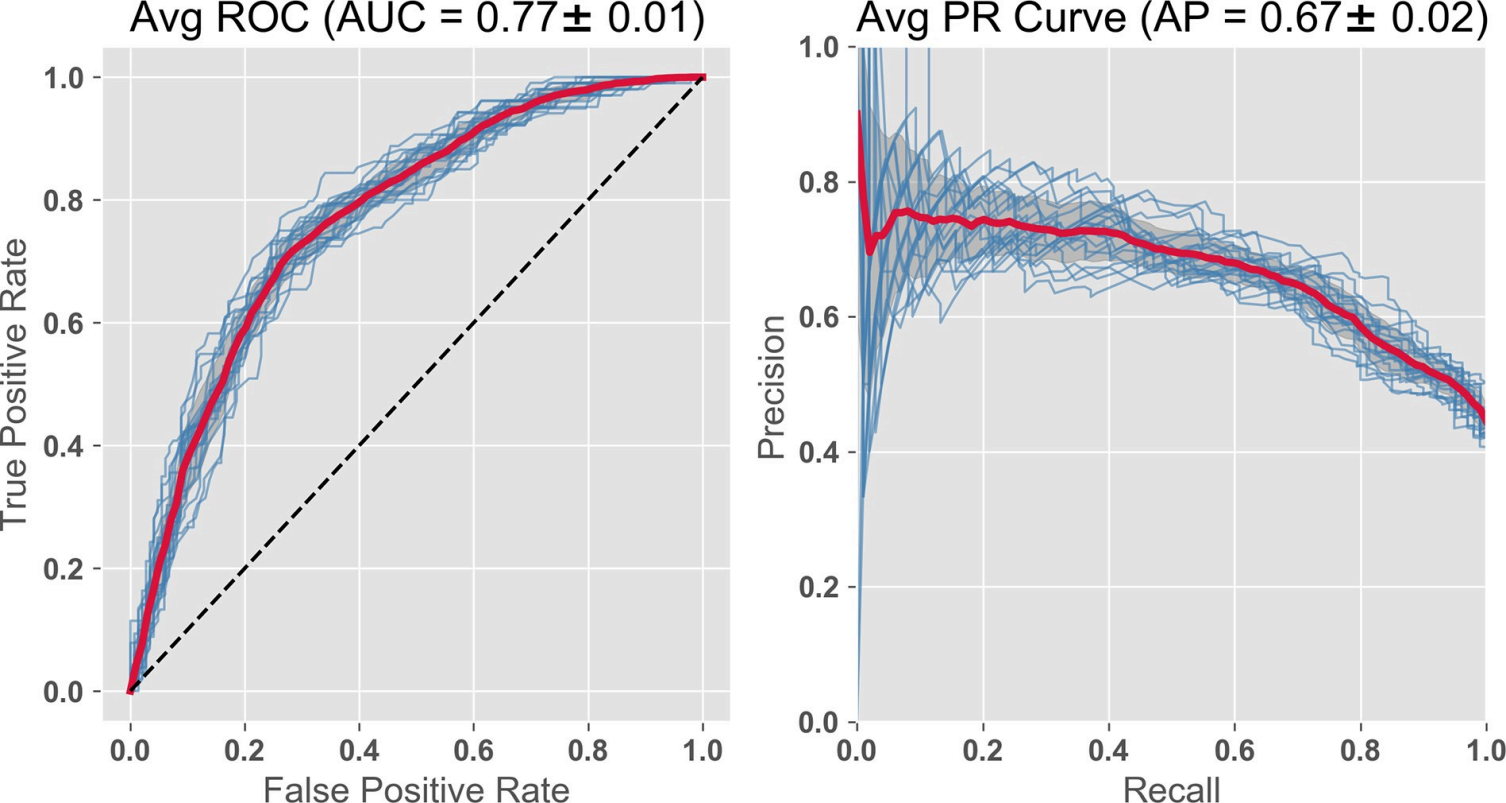
Further AUC ROC improvement is seen combining pBM, MRI, cognitive scores, atrophy rate and demographics. To the features used in Fig 6, we add 19 atrophy features (APC), estimates of annual % change. This improves the AUC from 0.75 to 0.77. The specific MRI regions of interests used to generate atrophy variables are shown in Table 3 where provided is the ADNI code, the neuroanatomical name, and GINI coefficient weighting.

Since the cortical atrophy variables are known to be important as AD predictors [14], we added 19 APC (annual percent change) atrophy variables to the feature set just used. The AUC value improved to 0.77 (see Fig 7). The precision recall (PR) curves also improve over this same sequence (Figs 4–7). As AUC increases from 0.71 to 0.77, the PR curves increase from 0.60 to 0.67. However, acquiring the atrophy features requires a second imaging session, thus adding to cost. The improvement is to some extent expected, since we are getting a peak further into the future and closer to the predicted event. In any case, using non-invasive and relatively inexpensive testing including cognitive, genetic risk, age, and, for example, kit inflammatory plasma biomarkers with Random Forest (or Gradient Boost) gives a high AUC of 0.71, which we saw improved slightly to 0.73 by substituting MRI data at baseline (and further improved to 0.75 by combining both modalities).

From Table 3 GINI coefficient data of the features, we can assess importance. We note that among the top 5 features are the 2 ADNI cognitive performance variables, the plasma analytes Apolipoprotein E and C Reactive protein, as well as right hippocampal atrophy rate.

**Table 3:** GINI importance of features from plasma BMs, MRI, cognitive, atrophy rate & demographics.

ADNI Code	Variable	Weight	ADNI Code	Label	Weight
ADNI EF	Executive Function	0.0717	ST50TA	APC; L. Posterior Cingulate	0.0247
ApoE (ug/ml)	Apolipoprotein E	0.0571	ST109TA	APC; R. Posterior Cingulate	0.0216
ADNI Mem	Memory	0.0526	Apo Aii (ug/ml)	Apolipoprotein Aii	0.0205
CRP (ug/ml)	C Reactive Protein	0.0379	Apo Ciii (ug/ml)	Apolipoprotein Ciii	0.0202
ST88SV	APC_R. Hippocampus	0.0351	ST19SV	APC; L. Gray Matter Cortex	0.0199
ST79SV	APC_R. White Matter_Cortex	0.0324	ST37SV	APC; L. Lateral Ventricle	0.0198
ST52TA	APC; L. Precu-neous	0.0302	ST12SV	APC; L. Amygdala	0.0196
MRI EV #4	Eigenvector	0.0297	ST29SV	APC L. Hippo- campus	0.0196
ST111TA	APC; R. Precu-neous	0.0286	ST78SV	APC; R. Gray Matter cortex	0.0191
Apo D (ug/ml)	Apolipoprotein D	0.0271	Apo Ai (ug/ml)	Apolipoprotein Ai	0.0191
ST24TA	APC; L. Entorh-inal Cortex	0.0266	Age	Age (yrs)	0.0189
ST83TA	APC; R. Entorh- inal Cortex	0.0260	Apo B (ug/ml)	Apolipoprotein B	0.0189
BMI	Body Mass Index	0.0250	Insulin (U/ml)		0.0188

Features by ADNI code in the first column, and then by standard name in the second column, where “APC” indicates annualized atrophy rate as a percent–rather than baseline value.

In summary, predictions of a transition from MCI to AD using Random Forest techniques depend strongly on baseline values of cognitive performance. Adding either plasma biomarkers or MRI alone increased AUC values further above 0.7, and combining them increased AUC further to 0.75. Addition of atrophy variables leads to an even higher AUC level, ~0.77, an improvement that comes at the economic cost of additional MRI sessions.

### Feature importance

Given the large number of features, it is useful to rank their classifying potential, thus giving a rational basis for prioritizing data collection in future studies. Random Forests have a built-in method for calculating feature importance in relation to a given classification task [29] (Chapter 6). This is obtained by comparing each variable’s ability to decrease the GINI impurity metric across the trees in the forest. The tables above (I-III) have feature lists ordered by importance based on this metric. While some features are often seen at the top of the list, the exact ordering of particular features is not standard. Some important features for one group may not maintain that importance when combined with a different group of features.

We asked the question here whether the order of importance remains relatively consistent if the feature set is maintained, but we examine a different random group of test subjects. While some of the order is maintained, the absolute values of the Gini coefficients and the AUC vary as different random groups of test subjects are chosen. So in our figure presentation above, we have used averages of the 20 subject cohorts (Figs 2 and 3), and/or have shown results from each subject cohorts overlapped as individual lines (Figs 4–7).

The potential utility of lowering the number of features from the raw total (~500) to values much smaller than the number of subjects is apparent. The cognitive variables were already compressed by ADNI from a few dozen test results to two parameters. The several hundred baseline MRI variables (MRI1) were compressed to 5, where 2 of the eigenvectors partially represent particular physical (e.g. temporal lobes, fluid spaces) and functional attributes (executive function, memory scores). Decent AUC values were attained during predictions with as few as 7 features.

To find subgroups for the ~150 plasma biomarkers in ADNI, we could pick analytes from literature reports studying Alzheimer’s or categories incidentally identified commercially (biotech testing companies for example). There are different Kits available from Rules Based Medicine (RBM), the company which did the testing for ADNI, and each has its own list of analytes which we used in our model. For one particular line of inquiry, we examined the reproducibility as a function of subject cohort. First, we concentrated on feature groups generated with non-invasive testing and without imaging. This included the feature groups cognitive performance, demographics, genetic risk (APO E4), and one of the plasma kits (kit metabolic or kit inflammatory). Then for contrast, we also looked at the most comprehensive group, the All Features Group. For all 3 examples, we had surprisingly consistent lists of feature importance order comparing several subject cohorts, picked at random.

**Fig 8:**
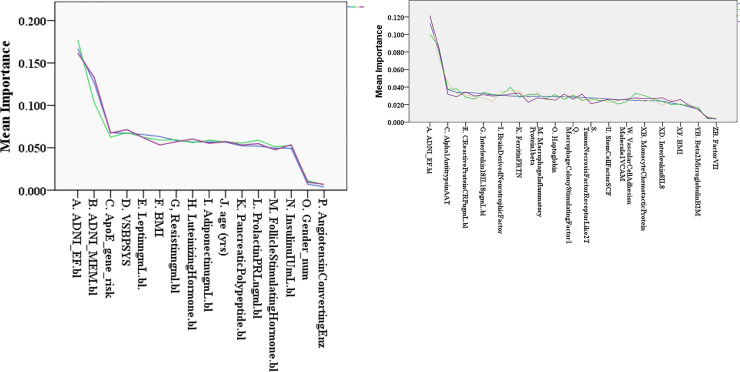
**a & b** represent plots of the importance coefficients (y axis) ordered on the X axis by feature importance. We used the mean importance from 3–4 subject groups, but the same features for each. Each line represents a different subject group. Top panel [a] is for the groups described above including Kit Metabolic and Panel [b] is for same plus Kit Inflammatory. The average ordering is shown. The general profile of the GINI coefficients is illustrated by the plots.

The next two figures (Fig 8A & 8B) use importance lists for several different groups of subjects. We used the reduction in the mean GINI impurity metric to generate an ordered list of importance (x axis), and then plotted the coefficients (y axis) for each cohort. Although, there was some disparity in terms of features switching places, or shifting up and down several places on the list, the correlations amongst the lines in the two figures was excellent. In Fig 8A., looking at 3 cohorts, we obtained an average correlation coefficient of 0.9829 ± 0.044 (mean ± SE; p<0.0005). The plasma biomarkers cohort was kit metabolic. In Fig 8B., the average correlation coefficient over all pairs of curves is 0.9702 ± 0.0033 (u, SE; p<0.0001). A similar exercise with the ALL FEATURES (n = 522) cohort also showed high correlation (0.702; p<0.0005).

In the importance lists from the All Features group from two different subject cohorts (i.d.,406505; i.d.,112315), we identified the features that occurred in the first 25 slots for both, i.e. the number of pairs among the 25 best features. There were 16 pairs (features found in both subject cohorts), including plasma analytes alpha1 micro-globulin, alpha2 macro-globulin [30], vitronectin, follicle stimulating hormone, plus intracranial volume, atrophy rate of right sided cortical gray matter, choroid plexus, and portions of the following brain regions (cingulate, entorhinal cortex, frontal gyrus), plus right inferior lateral ventricle volume. Of these 16 features constituting the pairs, 13 were in the list of top 25 features generated from an average over all 20 subject cohorts. The feature groups for the non-invasive, non-imaging features were cognitive performance, demographic, ApoE4 risk and Kit Metabolic (panel a); and the top 10 predictors were ADNI EF, ADNI Mem, VSBPSYS (systolic blood pressure), ApoE4 gene risk, Leptin, Adiponectin, Resistin, Age, Luteinizing hormone, and prolactin. For the 2^nd^ feature group (cognitive, demographic, ApoE4 risk, Kit inflammatory; panel b.) the best features were: ADNI EF, ADNI Mem, alpha 1 antitrypsin, SYS BP, Interferon gamma, BDNF, Macrophage colony stimulating factor, Von Willebrand Factor, and Ferritin.

#### Using GINI importance coefficients with Wilcoxon to rank importance of feature groups

We now compare importance for plasma biomarkers and MRI feature groups (of comparable size). We used GINI importance coefficients to rank individual features and then Wilcoxon non-parametric techniques to determine which modality (feature group) had the highest importance. We compared Kit Inflammatory (n = 26) with MRInorm (n = 18) and separately with MRI2 (n = 18); Kit Neuro (n = 24) with MRI2; and, total plasma biomarker group (n = 143) with MRI1 (n = 330).

Kit inflammatory had a higher mean rank than MRInorm (26.8 vs 16.3; p = 0.008); and, than MRI2 (27.8 vs 15.0; p = 0.001). Kit neuro had a higher rank than MRI2 (25.9 vs 16.7; p = 0.008). Finally, comparing 143 plasma biomarkers to 330 MRI ROIs derived a larger mean rank (258.15) for total plasma than MRI (227.83) at p = 0.027. This showed that plasma groups were very competitive, if not superior to, MRI feature groups using GINI coefficients.

#### Comparison of ROC AUC as a function of adding plasma features to other non-imaging features during disease progression

Looking at RF generated AUC values for ROC in 10 random test/train cohorts, we examined whether adding plasma to cognitive/demographic feature sets was a significant improvement. We also compared adding plasma features with adding features from MRI data. The best plasma group ‘hot set’ was compared against the features identified in various commercial kits. Starting with a baseline of cognitive and demographic features, we found that genetic risk and hot set both increased average AUC (p<0.05), with the combination further increasing AUC (p<0.01). However, adding kit cardiac or kit inflammatory alone was not significant (p<0.1). Starting with cognitive, demographic and genetic risk, AUC was increased by addition of plasma “hot set” (p<0.05). Certain combinations of adding MRI to cognitive and demographic were also effective to a similar extent as adding plasma “hot set”, namely: MRI1 (p<0.05), and MRIPCA (p<0.01). This is consistent with Figs 4–7 where combining features from imaging, cognitive, and plasma modalities saw step wise increases in AUC over the range of 0.7 to 0.8.

#### Comparing plasma and MRIPCA

In Table 4, we examine the Inflammatory Kit Biomarkers plus the compressed baseline MRI (MRI PCA), absent the powerful cognitive features which often dominate. We are left with a sparse feature set, a slightly different mix of modalities than prior runs; one which would be appropriate for an imaging center without resources for cognitive testing. Two of the features in the top 5 include 2 cytokines (TNFalpha; Interleukin 8) whose elevation has already been reported associated with post surgical cognitive loss [28].

**Table 4:** GINI importance features from MRI PCA and kit inflammatory.

Feature	Weight	Feature	Weight
Tumor Necrosis Factor alpha	0.09097	Von Willebrand Factor	0.03095
Vascular Cell Adhesion Molecule	0.07858	Fibrinogen	0.03071
MRI Eigenvector #5*	0.04592	Factor VII	0.03031
Interleukin 18	0.03466	Eotaxin1	0.03022
Interleukin 8	0.03446	C Reactive Protein	0.03007
Alpha1 Antitrypsin	0.03386	Brain Derived Neurotrophic Factor (BDNF)	0.02978
Beta2 Microglobulin	0.03129	VascularEndothelialGrowthFactor (VEGF)	0.02975
Tumor Necrosis Factor Receptor 2	0.03128	Stem Cell Factor (SCF)	0.02940

*Includes various areas from cingulate cortex.

#### Automatic diagnosis using plasma features at the point of disease inception–detecting deviations from normal

**Fig 9:**
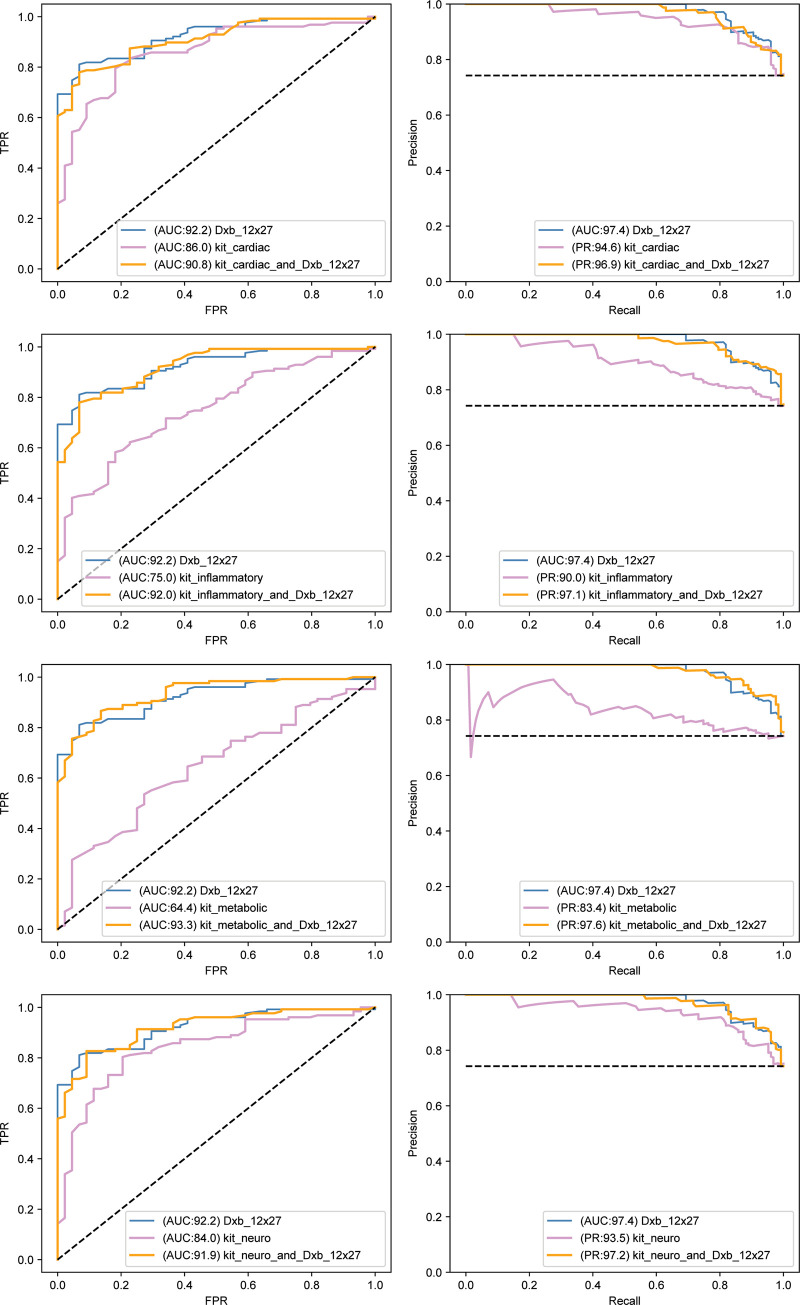
Left panels are Receiver Operator Curves (ROC) and right panels are associated precision recall curves. Blue curves are for the feature set comprising the putative best plasma feature cohort. Purple curves are for the feature set we are comparing,.and orange curves are for the combination of both feature sets. The axes for the ROC plots show true positive rate (TPR; y axis) versus false positive rate (FPR; x axis). The x-y axes for the ROC curves are recall (x) vs precision (y). The putative best plasma cohort is dxb_12x27. The comparators are kits cardiac, inflammatory, metabolic, and neuro. Results confirm that dxb_12x27 is the best feature group. AUC values are normalized to 100 (i.e. percent) in Figs 9 & 10.

**Fig 10:**
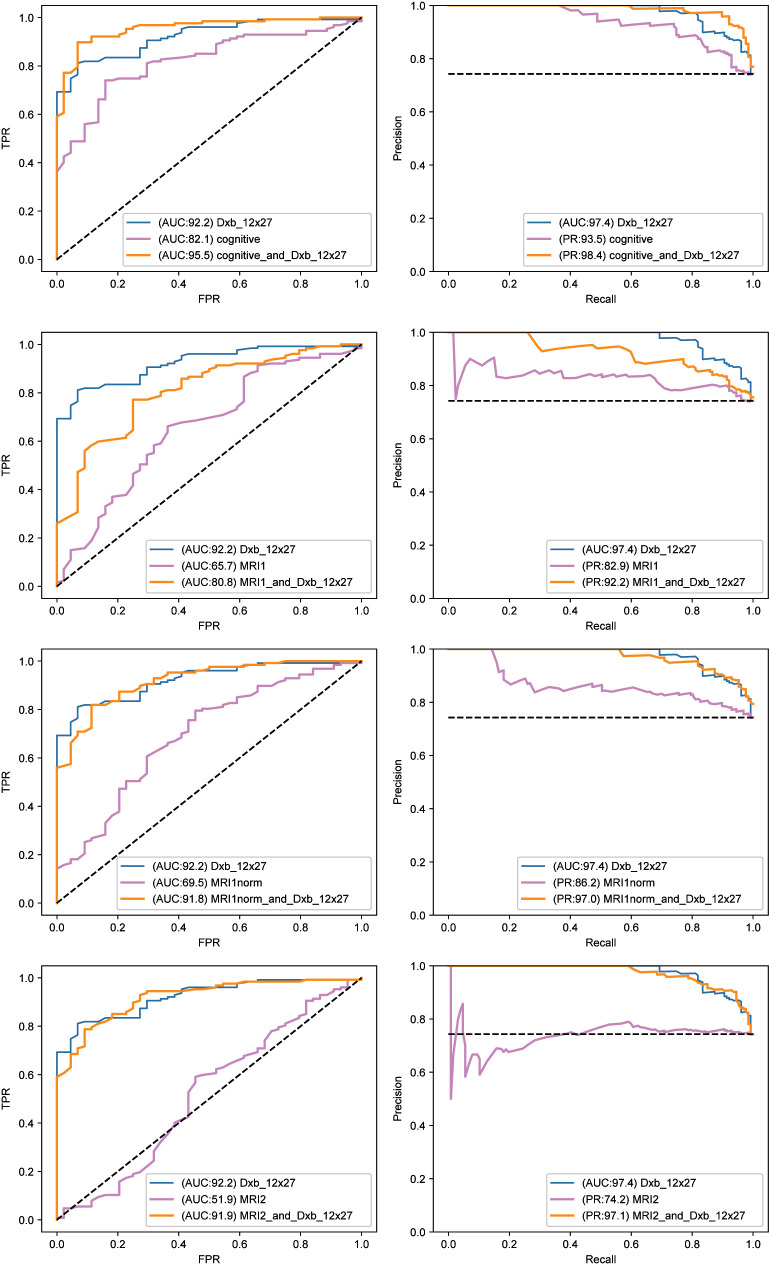
Comparison of best plasma feature group with different MRI and COG feature groups as comparators. The compactor feature groups are cognitive, MRI1, MRInorm, and MRI2 (from top to bottom). They are all compared to the best plasma feature group. Fig 10 has the same format as Fig 9.

The analysis of the earliest changes, as one moves out of diagnostic normal, was tested for a similar group of features as used for studying the later transition highlighted in Figs 4–7. We used Random Forests, shown to be very effective in the earlier analysis. The results are displayed in two figures which use (for each feature set) ROC AUC (left panel) and Precision/Recall curves (right panel). Fig 9 shows results from a variety of plasma biomarker groups, whereby the most effective was determined (namely dxb_12x27). In Fig 10, we compare this most effective plasma biomarker group (dxb_12x27) against 3 of the MRI representations and cognitive performance. Note the plasma biomarkers alone gave excellent AUC values, and, furthermore, the plasma biomarkers alone were competitive with or better than the MRI representations alone or cognitive performance alone. Adding the best plasma feature set improves the other modalities (MRI, COG).

Examining 5 different plasma cohorts (dxb_12 (best set), kit cardiac, kit neuro, kit inflammatory and kit metabolic) in testing our ability to discriminate between NL and early MCI, we found that the best (dxb_12) had an AUC of 92.2 with the other plasma cohorts at AUC values 86.0, 84.0, 75.0, and 64.0. All had precision recall above 90.0 except Kit Metabolic (83.4). These were all better than predictors of progression from MCI to full AD (Figs 4–7), where the best AUC value was ~ 0.8 (80.0 normalized to 100).

We then compared the select dxb_12x27 plasma cohort with the full set of cognitive features and 3 MRI representations. Examining the features separately we obtained 92.2 (Dxb_12x27), 82.1 (Cognitive), 69.5 (MRInorm), 65.7 (MRI1) and 51.9 (MRI2). Adding the plasma cohort to cognitive or the MRI representations respectively gave AUC values of 95.5, 91.8, 80.8, and 91.9. All showed improved prediction (increased negativity in Z value) with addition of the plasma cohort and using nonparametric rank statistics (see Table 5).

**Table 5:** Statistics for plasma features alone & cognitive alone and paired with best plasma feature.

	Dxb_12x27	cognitive_alone	cognitive_and _Dxb_12x27	kit_cardiac_ alone	kit_inflamma-tory_alone	kit_metabolic_alone	kit_neuro _alone
Mann-Whitney U	437.500	1001.500	250.500	784.500	1396.000	1990.000	893.000
Wil Wilcoxon W	1427.500	1991.500	1240.500	1774.500	2386.000	2980.000	1883.000
ZZ Z	-8.327	-6.362	-8.988	-7.101	-4.940	-2.841	-6.718
Asymp. Sig. (2-tailed)	.000	.000	.000	.000	.000	.004	.000

Goodness of discrimination of NLs from stable MCI using RF techniques. The putative best plasma cohort (Dxb_12x27) is better than Cog alone, or MRI feature groups, or other plasma groups based on p and Z values.

All the plasma feature cohorts alone as well as cognitive alone were significant at p = 0.000 showing the same order in AUC values (dxb_12x27, kit cardiac, kit neuro, kit inflammatory, and kit metabolic) as negative Z values. Cognitive was comparable to kit neuro, and smaller than dxb_12x27 (which when added to it increased the negative Z value). The best plasma cohort had better Z values than any of the MRI feature groups alone, and Improved their predictions when combined with them (see Table 6).

**Table 6:** Statistics for MRI groups alone & paired with best plasma feature group.

	MRI1_ alone	MRI1_and_ Dxb_12x27	MRI1norm_alone	MRI1norm and_Dxb_12x27	MRI2_alone	MRI2_and_ Dxb_12x27
Mann-Whitney U	1914.000	1073.000	1705.000	458.500	2686.500	452.500
Wilcoxon W	2904.000	2063.000	2695.000	1448.500	3676.500	1442.500
Z	-3.110	-6.082	-3.848	-8.253	-.380	-8.274
Asymp. Sig. (2-tailed)	.002	.000	.000	.000	.704	.000

Goodness of discrimination of NLs from stable MCI using RF techniques. The putative best plasma cohort (Dxb_12x27) is better than Cog alone, or MRI feature groups, or other plasma groups based on p and Z values.

## Discussion

For Alzheimer’s Disease, early detection is crucial and often occurs in the primary care setting, a setting lacking in specialized imaging equipment [31]. Although there are many points where deviations from normal could result in interpretable biomarker signals, the anatomical distribution of the source of these analytes, the measurement noise, the variability due to factors only marginally related to AD risk (e.g. normal aging, coronary artery disease), as well as collinearities amongst biomarkers, present challenges in developing explicit biomarker signatures with generalizable relevance over many patients.

However, our work has shown that a few inexpensive biomarkers, using random forests or gradient boosting approaches, can give results that are nearly as good as using data from more expensive imaging techniques like MRI and PET, which however should be used as a final validator. We also note that PET imaging and CSF sampling from the spinal cord are highly predictive; but are considered ‘greater than minimal risk’ interventions by human subject regulations. Relatively inexpensive predictions could be based on analyzing milliliter volume blood samples for multiple peptide analytes, genetic mutations with further improvements from cognitive testing—all of which are non-invasive. These less expensive, less invasive biomarkers also happen to consume far less patient and staff time during the acquisition step, allowing for mass screening and participation from sites with modest resources. A comprehensive discussion of all the ways variables may predict the progression of Alzheimer’s are beyond the scope of this article, and have been reviewed many times. However, we briefly touch on these categories in the S3 Appendix section with particular emphasis on the different groups of plasma biomarkers. Lists of plasma biomarkers included.

### Clinical application of approach

We consider the Alzheimer’s Association protocol for managing and diagnosing AD in order to understand how the analysis described in this research could be applied. In summary, an initial self-report of cognitive problems leads to a variety of rule-outs whereby alternative causes are assessed, treated as warranted and/or discarded as options. If there is no improvement in cognitive behavior, then the next cycle is engaged. Eventually one confirms AD or an alternative diagnosis. For example, one might initially look for potential pharmacological side effects, responding by adjusting medication dosing. Systemic illness can be postulated, leading to tests for (and potentially treatment of) hypothyroidism and B12 deficiency, for example. If there is still no improvement in cognitive status, then imaging (CT or MRI) is used to rule out other dementia causes like vascular dementia, hydrocephalus, tumors, and subdural hematoma. If there are signs of depression, it is treated. If still no improvement, Alzheimer’s remains the leading diagnosis.

How could we employ our findings to treat Alzheimer’s? If an important feature involved in predicting progression turned out to be relatively elevated, then blocking or buffering the biomarker analyte could be therapeutic in the event that the elevation were causal, not just symptomatic. For example, if the deviation of a feature were toward below average values, and also causal, diet supplementation could be considered and tested. The machine learning analysis we outlined could be employed at a variety of time points, or clinical milestones; and changes in biomarkers could augur changes in progression.

Different panels of biomarkers could be used at different times in the disease progression, as suggested by our results indicating stronger prediction from plasma biomarkers at disease inception. We have shown the miscibility of the different types of features (anatomical features, plasma analytes, gene mutations, cognitive performance, demographics) which can be incorporated in the same analysis. As shown in the studies of Jack et al. [17, 32], detectable changes of variables from different modalities often follow sigmoidal curves over time and indicate the steps of progression.

### Use of machine learning techniques

Attempts have been made to identify ADNI elements for predicting disease inception [33] and/or progression [34] based on diagnostic biomarkers from various modalities. The utility of multiple modalities has become clear, although researchers had initially specialized in single modality studies [35]. Yet, analyses from single modalities have also been productive, especially when combined with advanced techniques like Random Forests and as applied to complex modalities like structural MRI. Successful attempts have been reported discriminating between, for example, 4 different diagnoses (healthy controls, early MCI, progressing MCI and AD) with one modality, MRI [34].

Various procedures have been used to compress features within a modality, or multiple modalities. This “early fusion” [34] can occur prior to use of machine learning techniques. Alternatively, “late fusion” can combine post ML the derived variables [34]. Among the most popular ML techniques are SVM, which we found worked well with lightly populated cohorts and RF [34, 35], the overall best of the 4 ML approaches we employed [36].

MRI ROIs with various units like cortical volume, thickness or area can be normalized by intracranial volume, age or stratified by categoricals like gender or genetic risk [37]. MRI variables can be described in terms of rate of change (atrophy); or compressed into fewer variables using principal component analysis (PCA) [38]. RF produces the GINI importance coefficients available to help select a smaller group of important features [39]. Preparation of our four MRI feature sets used many of these approaches. Finally, outcomes can be converted from continuous variables into categorical variables like diagnostic labels using cutpoints [37].

Among the more novel findings in this current study are that the best features for prediction tend to shift during disease progression, whereby plasma analytes are more powerful at disease inception, and MRI later. Furthermore, we have highlighted the often overlooked plasma analytes and shown their potential utility at the point of disease inception. Since much of the research being done on blood biomarkers focused on a few analytes at a time, we hypothesized that machine learning techniques could allow for a broad examination of many features simultaneously Standard problems with CART, like overfitting, can be resolved by using more advanced approaches designed to counter these problems, e.g. random forests or gradient boost techniques. This advantage was clear in Figs 2 & 3, where CART was consistently inferior.

By using simple blood tests in combination with machine learning, we may be able to identify patients at risk and/or patients who would benefit from additional testing, thereby motivating and justifying more costly and invasive tests. This allows us to approach prediction along a less expensive and lower risk path. Resulting hypotheses can be confirmed using more standard diagnostics (like MRI, PET or CSF sampling of amyloid beta and phosphorylated tau). Being able to use non-invasive, convenient, and relatively inexpensive tests allow for pre-symptomatic tracking of large groups facilitating both early diagnosis, and FDA drug testing because it would allow studies to track changes in the predictions after treatment. The targeted clinical transition explored in Figs 2–7 is a little late in the disease process, but nonetheless very important in identifying patients at imminent high risk for disabling advanced dementia. The later analysis in this report (Figs 9 & 10), using automatic diagnosis at disease inception, does focus on early detection and identifies a feature group that is quite appropriate for population screening that would be required at this clinical milestone.

## Supporting information

S1 Appendix (DOCX)

S2 Appendix (DOCX)

S3 Appendix (DOCX)
