# Supplementary material for: Inexpensive, non-invasive biomarkers predict Alzheimer transition using machine learning analysis of the Alzheimer’s Disease Neuroimaging (ADNI) database
Source: PLoS One. 2020 Jul 27;15(7):e0235663. doi: 10.1371/journal.pone.0235663 (PMC7384664; doi:10.1371/journal.pone.0235663)

**S1. Appendix: Inexpensive Non-invasive Biomarkers Predict Alzheimer Transition**

**Using Machine Learning Analysis of the**

**Alzheimer’s Disease Neuroimaging (ADNI) Database**

- **Schematic Comparing Standard Decision tree classifier from random tree classifier as used in Random Forest.**

**Figure: Starting from a set of all patients, a decision tree classifier (left) queries all available features to maximize the purity of the nodes generated when splitting on that feature (ST31TA <1.935). Alternatively, in a random tree, the splitting procedure can query only a subset of the features, chosen at random, to enhance the diversity of the trees. In this schematic, the random tree is allowed to query only one random feature per node. Nodes in figure below are described along with units. ADNI code for cortical thickness (in mm) is Left Inferior Parietal (ST31TA) and Left Precuneus (ST52TA); for surface area (mm^2^) of Left Entorhinal Cortex (ST83SA); for Std Dev of cortical thickness (mm) of left parahippocampal (ST44TS); and for volume (mm^3^) of left hippocampus (ST29SV). Units for cortisol and testosterone values are ng/ml.**

**S1 Appendix Fig. A.**


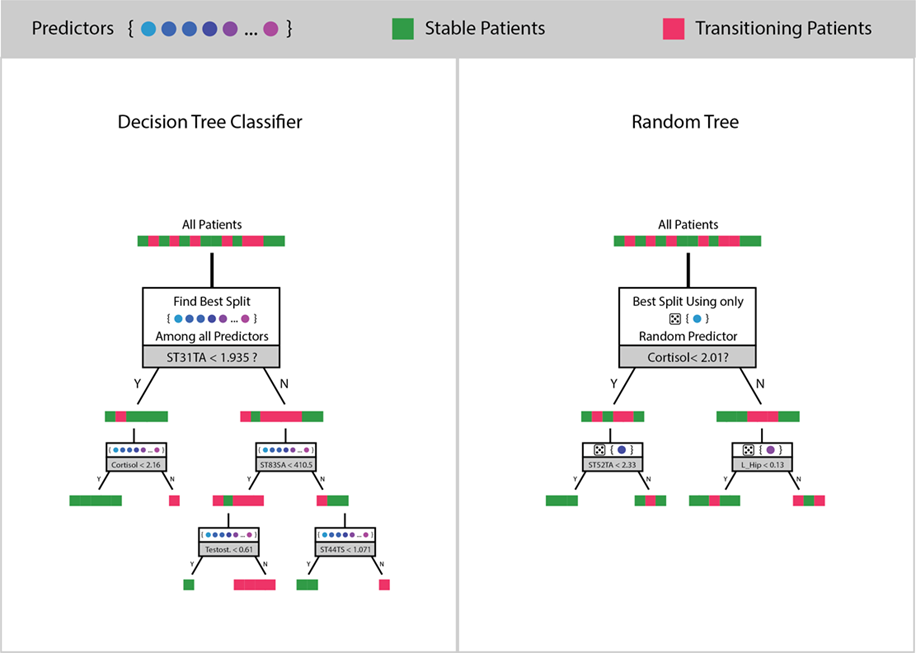

Supplement: S1 Appendix — (DOCX) [file pone.0235663.s001.docx]
