# Supplementary material for: Inexpensive, non-invasive biomarkers predict Alzheimer transition using machine learning analysis of the Alzheimer’s Disease Neuroimaging (ADNI) database
Source: PLoS One. 2020 Jul 27;15(7):e0235663. doi: 10.1371/journal.pone.0235663 (PMC7384664; doi:10.1371/journal.pone.0235663)
Supplement: S2 Appendix — (DOCX) [file pone.0235663.s002.docx]

**S2. Appendix: Inexpensive Non-invasive Biomarkers Predict Alzheimer Transition**

**Using Machine Learning Approaches in**

**Analysis of the Alzheimer’s Disease Neuroimaging (ADNI) Database**

- **Comparison of CART analysis versus Random Forest analysis for a comprehensive set of features, identical for each method.**

**Performance using CART –**

The first analysis used standard CART to predict subject transitions; but was unsuccessful, as shown in the two figures (Figs. 2 & 3). The following section compares CART and Random Forest (RF) in more detail.

The TPR/FPR graph, also known as the Receiver Operating Characteristic (ROC) curve, is a measurement of a metric's ability to discriminate between two classes. The area under the ROC curve (AUC ROC) serves as a measurement of the correlation between the prediction and the true labels across the dataset, where 0.5 is the expected AUC ROC for random classifier performance (1). The specific features included here were: plasma biomarkers (pBM), atrophy (APC) features, static, baseline MRI values expressed as individual ROIs, compressed into eigenvectors, and with values normalized to intracranial volume; demographics and, the cognitive variables (ADNI_EF and ADNI_Mem). For this extensive list of almost 500 features, the analysis was not effective, with AUC = 0.59 for the ROC value (see Fig. A, left panel.). Without the two cognitive features, the results were even less predictive, AUC = 0.045.

**Random Forest Classifier Produces Better Results Than CART**

As indicated decisively in Figs 2 and Fig. 3, Random Forests perform well in high-dimensional spaces, as the random picking of features to test at each node captures non-linear as well as linear interactions. What follows is a Random Forest analysis of the same feature set as above for CART, resulting in much improved precision recall values, and a reasonable value, AUC = 0.73, for the ROC curve (see right panel). All subsequent comparisons of the utility of different feature groups will use Random Forest as the chosen ML approach.

**S2 FIG. A.**

All features: CART (Left panel) and RANDOM FOREST (Right panel)


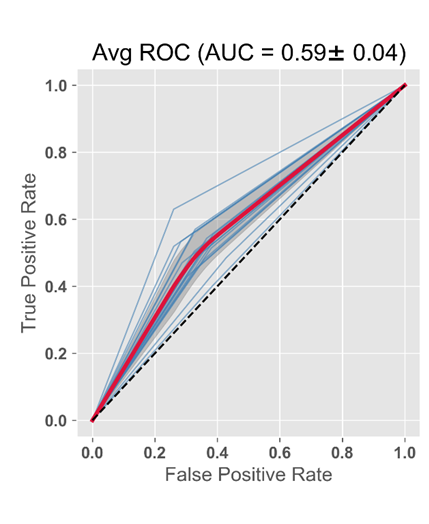

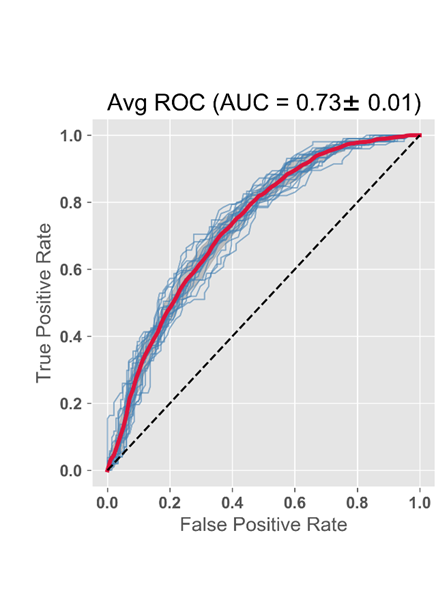


1. Power DM. Evaluation: From Precision, Recall and F-Measure to ROC, Informedness, Markedness and Correlation. Journal of Machine Learning Technology. 2011;2:37-63.
