# Supplementary material for: Inexpensive, non-invasive biomarkers predict Alzheimer transition using machine learning analysis of the Alzheimer’s Disease Neuroimaging (ADNI) database
Source: PLoS One. 2020 Jul 27;15(7):e0235663. doi: 10.1371/journal.pone.0235663 (PMC7384664; doi:10.1371/journal.pone.0235663)
Supplement: S3 Appendix — (DOCX) [file pone.0235663.s003.docx]

**S3. Appendix: Inexpensive Non-invasive Biomarkers Predict Alzheimer Transition using**

**Machine Learning Analysis of the Alzheimer’s Disease Neuroimaging (ADNI) Database**

- **Discussion of Features and Potential Relevance for Diagnosing and Predicting Alzheimer’s Progression.**

**Available Features and Their Relevance**

The features that seemed useful as part of a collection were genetics (i.e., the ApoE4 mutation), MRI (using several representations of baseline anatomy, as well as longitudinal rate of change – atrophy rate), demographics (age, BMI, gender, blood pressure), and plasma biomarkers.

Obviously, neural survival is a result of many biological pathways which deliver oxygen and nutrients (cardiac), allow for its use (metabolic), protect neurons from pathogens (inflammatory) but damage them if not regulated well. Neural homeostasis depends on the interplay of many cortical environmental factors controlling biological health, and also the effect of vigorous neural activity resulting from brain waves and cognitive behavior. The physical entities we chose as biomarkers are both upstream and downstream of the pathological processes underlying AD as well as of healthy brain activity. The general classes of biomarkers we examined are discussed below.

*Magnetic resonance imaging* – Our analysis to the complete set of several hundred ADNI ROIs routinely identified as changed many of those areas considered key to the path of neural atrophy in AD progression. Examination of the compressed representation of the MRI ROIs, generated by principal component analysis, identified the eigenvectors we had previously shown to strongly correlate with ADNI compressed cognitive performance: particularly, one associated with executive function and one with memory (1). The third representation for the MRI normalized for skull size, although not as effective by itself, had unique utility when paired with other features. The variable describing the intracranial volume itself was identified as an important feature when using a non normalized MRI representation. The atrophy features, especially those in the temporal lobes, were very powerful – as to be expected from the literature.

*Genetics* *-* Genetics play an important role in the development of AD. Autosomal dominant AD is characteristic of specific biomarkers and symptoms, some of which biomarkers can be measured up to two decades before the appearance of symptoms (2).

Several apolipoproteins (APO) are associated with AD, and appear frequently as important features in this study (especially APO Aii, Aiv, Ciii, E and J). Alleles of these proteins impair the functioning of astrocytes and microglia in clearance of amyloid beta (3). Elevated clusterin (APO J) impairs neural efficiency (4), working memory (5), and leads to cognitive decline (6) in patients with AD (7), as well as in those patients with adiposity and components of metabolic syndrome (8).

The APO E4 gene allele confers a strong risk for AD via brain atrophy (9) and the pathological aggregation of amyloid beta (10). Various single nucleotide polymorphisms (SNP) are also risk factors (11). Due to the strong dependence of normal functioning of complex protein structure and the chaperone system, on both energy and suppression of sequence errors, the risk for inappropriate amyloid aggregation is high where certain mutations occur.

As clear from above, amyloid plaques formed by amyloid beta, and neurofibrillary tangles due to hyperphosphorylated tau protein (12) are thought to be involved in AD progression. Although the associated biomarkers are included in ADNI, the number of subjects for which this data is available is limited. This lack of overlap and small number of subjects was the case also with positron emission tomography (PET). So, we do not report on these variables here.

*Plasma biomarkers* – in this section, we review literature that demonstrates well-documented patterns between plasma biomarker changes and onset or progression of AD. This is to point out that the relation of AD to certain biochemical pathways is already known, and based on measurement of changes in these pathways prior to or subsequent to AD inception or progression. We did not need the Machine Learning techniques to show the biomarker changes associated with AD, just to show that these changes can be used to predict AD progression and that combining the dynamics of a multitude of biomarkers can assist in doing this. Early in the project, we used a set of plasma biomarkers picked from the literature, but later relied on the categories used by a major commercial test facility. This reduced potential bias by the authors, was helpful mechanistically since the kits fit in well with the etiology of the disease, and gave a more manageable size test group. The kits were: metabolic, inflammatory, neural, cardiac. We discuss a few examples of how salient features in these groups work. However, by combining the plasma features from the different kits with the highest GINI coefficients, we did obtain a better single set of plasma BMs (i.e. the greatest hits collection; which included, amongst others, apolipoprotein Aii, Aiv, E; vitronectin, C reactive protein, leptin, CD40 ligand, beta 2 microglobulin, TNF alpha, matrix metalloproteinase 7, etc).

*Kit Metabolic* ***–*** Various metabolic regulators and substrates affect neural proliferation and survival. Decreased signaling of the metabolic hormone leptin has been associated with cognitive decline (13, 14); and is suggested to protect against accumulation of amyloid beta (15) by inhibiting it on the surface of hypothalamic neurons (16, 17). Previous analysis of the ADNI database has shown a well defined positive correlation of leptin and BMI, where this entire curve is shifted toward higher leptin for females. However, leptin also positively correlates with brain volume, consistent with the fall in leptin during AD progression (18, 19). Yet, some suggest that leptin alone is insufficient to ascertain AD (20). Yet, in these analyses, leptin (an analyte in the kit metabolic group) was flagged as an important feature.

Insulin, an important hormone regulator of glucose metabolism and also a member of the kit metabolic group, can malfunction - contributing to AD progression and acting as an early biomarker (21-23). Insulin’s regulatory targets also include amyloid beta (24). Thus, increased risk (~65%) of AD in diabetic patients is not surprising (22). Multifactorial conditions such as **metabolic syndrome**, are associated with hippocampal atrophy (25, 26), a common early feature in AD.

*Kit Inflammatory* ***–*** Various chronic conditions including obesity, and periodontitis, as well as other causes of systemic inflammation, can lead to an increase in blood analytes including CRP, interleukins (IL6, IL8, IL1), IFN-gamma, cortisol, TNF-alpha (25), and other proinflammatory cytokines. This persistent inflammatory milieu can accelerate cognitive decline (27), increasing the risk of AD (22, 28-30). In fact, enhanced inflammation may contribute to cognitive dysfunction in the months following surgery (31). CRP serum levels are associated with cognitive impairment (32); specifically, an increase in CRP was found associated with development of AD, and a decrease in CRP with established AD (33, 34) (19). These and other inflammatory biomarkers included in the Kit Inflammatory group of features were flagged as important in our results, especially CRP.

*Kit Neural, Kit Cardiac****-*** Reduced levels of serum brain-derived neurotropic factor (BDNF) are suggested to contribute to the pathenogenesis of AD (35), as BDNF is central to cell survival, dendritic spine formation, memory consolidation and synaptic transmission (36). Consistently, the Framingham Heart Study suggested that increased serum BDNF protect against dementia (37, 38), as do elevated levels of brain natriuretic peptide (BNP) (30) as well as atrial natriuretic peptide (ANP) (39-41). Thus, the relationship of many plasma biomarkers to neural processes and cardiovascular health has been established and can readily be detected. So, it remains to show that their interaction and change during complex processes can be used predictively.

1. Doan L, Choi D, Kline R, Alzheimers Disease Neuroimaging I. Impact of analgesics on executive function and memory in the Alzheimer's Disease Neuroimaging Initiative Database. Scand J Pain. 2017.

2. Bateman RJ, Xiong C, Benzinger TL, Fagan AM, Goate A, Fox NC, et al. Clinical and biomarker changes in dominantly inherited Alzheimer's disease. N Engl J Med. 2012;367(9):795-804.

3. Mulder SD, Nielsen HM, Blankenstein MA, Eikelenboom P, Veerhuis R. Apolipoproteins E and J interfere with amyloid-beta uptake by primary human astrocytes and microglia in vitro. Glia. 2014;62(4):493-503.

4. Lancaster TM, Brindley LM, Tansey KE, Sims RC, Mantripragada K, Owen M, et al. Alzheimer's risk variant in CLU is associated with neural inefficiency in healthy individuals. Alzheimers Dement. 2014.

5. Stevens BW, DiBattista AM, William Rebeck G, Green AE. A gene-brain-cognition pathway for the effect of an Alzheimers risk gene on working memory in young adults. Neuropsychologia. 2014;61:143-9.

6. Sattlecker M, Kiddle SJ, Newhouse S, Proitsi P, Nelson S, Williams S, et al. Alzheimer's disease biomarker discovery using SOMAscan multiplexed protein technology. Alzheimers Dement. 2014;10(6):724-34.

7. Wu ZC, Yu JT, Li Y, Tan L. Clusterin in Alzheimer's disease. Adv Clin Chem. 2012;56:155-73.

8. Won JC, Park CY, Oh SW, Lee ES, Youn BS, Kim MS. Plasma clusterin (ApoJ) levels are associated with adiposity and systemic inflammation. PLoS One. 2014;9(7):e103351.

9. Kim YJ, Cho H, Kim YJ, Ki CS, Chung SJ, Ye BS, et al. Apolipoprotein E4 Affects Topographical Changes in Hippocampal and Cortical Atrophy in Alzheimer's Disease Dementia: A Five-Year Longitudinal Study. J Alzheimers Dis. 2014.

10. Deroo S. Chemical crosslinking/mass spectrometry maps the amyloid beta peptide binding region on both apolipoprotein E domains. ACS Chem Biol. 2014.

11. Zhu XC, Tan L, Jiang T, Tan MS, Zhang W, Yu JT. Association of IL-12A and IL-12B polymorphisms with Alzheimer's disease susceptibility in a Han Chinese population. J Neuroimmunol. 2014;274(1-2):180-4.

12. Serrano-Pozo A, Frosch MP, Masliah E, Hyman BT. Neuropathological alterations in Alzheimer disease. Cold Spring Harb Perspect Med. 2011;1(1):a006189.

13. Tendas A, Cupelli L, Scaramucci L, Palombi M, Trawinska MM, Giovannini M, et al. Authors' reply. Indian journal of palliative care. 2011;17(3):260-1.

14. Bonda DJ, Stone JG, Torres SL, Siedlak SL, Perry G, Kryscio R, et al. Dysregulation of leptin signaling in Alzheimer disease: evidence for neuronal leptin resistance. J Neurochem. 2014;128(1):162-72.

15. Holden KF, Lindquist K, Tylavsky FA, Rosano C, Harris TB, Yaffe K. Serum leptin level and cognition in the elderly: Findings from the Health ABC Study. Neurobiol Aging. 2009;30(9):1483-9.

16. Yamamoto N, Tanida M, Kasahara R, Sobue K, Suzuki K. Leptin inhibits amyloid beta-protein fibrillogenesis by decreasing GM1 gangliosides on the neuronal cell surface through PI3K/Akt/mTOR pathway. J Neurochem. 2014;131(3):323-32.

17. Gomes S, Martins I, Fonseca AC, Oliveira CR, Resende R, Pereira CM. Protective effect of leptin and ghrelin against toxicity induced by amyloid-beta oligomers in a hypothalamic cell line. J Neuroendocrinol. 2014;26(3):176-85.

18. Kline RP, Haile M. Association of Leptin and Insulin with Cortical Atrophy in the ADNI Database. ASA Annual Meeting. 2013;Abstract Procedings.

19. Kline RP, Cuadrado FF, Haile M. Cortical Risk Factors Related To surgery From ADNI Database Blood and Imaging Biomarkers. Anesthesia & Analgesia. 2014;118(5S):S-151.

20. Oania R, McEvoy LK. Plasma leptin levels are not predictive of dementia in patients with mild cognitive impairment. Age Ageing. 2015;44(1):53-8.

21. de la Monte SM, Wands JR. Alzheimer's disease is type 3 diabetes-evidence reviewed. J Diabetes Sci Technol. 2008;2(6):1101-13.

22. Barbagallo M, Dominguez LJ. Type 2 diabetes mellitus and Alzheimer's disease. World J Diabetes. 2014;5(6):889-93.

23. Steen E, Terry BM, Rivera EJ, Cannon JL, Neely TR, Tavares R, et al. Impaired insulin and insulin-like growth factor expression and signaling mechanisms in Alzheimer's disease--is this type 3 diabetes? J Alzheimers Dis. 2005;7(1):63-80.

24. Fernandez-Gamba A, Leal MC, Morelli L, Castano EM. Insulin-degrading enzyme: structure-function relationship and its possible roles in health and disease. Curr Pharm Des. 2009;15(31):3644-55.

25. Nguyen JC, Killcross AS, Jenkins TA. Obesity and cognitive decline: role of inflammation and vascular changes. Front Neurosci. 2014;8:375.

26. Lin F, Lo RY, Cole D, Ducharme S, Chen DG, Mapstone M, et al. Longitudinal effects of metabolic syndrome on Alzheimer and vascular related brain pathology. Dement Geriatr Cogn Dis Extra. 2014;4(2):184-94.

27. Noble JM, Scarmeas N, Celenti RS, Elkind MS, Wright CB, Schupf N, et al. Serum IgG antibody levels to periodontal microbiota are associated with incident Alzheimer disease. PLoS One. 2014;9(12):e114959.

28. Kamer AR, Pirraglia E, Tsui W, Rusinek H, Vallabhajosula S, Mosconi L, et al. Periodontal disease associates with higher brain amyloid load in normal elderly. Neurobiol Aging. 2015;36(2):627-33.

29. Jabbari Azad F, Talaei A, Rafatpanah H, Yousefzadeh H, Jafari R, Talaei A, et al. Association between Cytokine Production and Disease Severity in Alzheimer's Disease. Iran J Allergy Asthma Immunol. 2014;13(6):433-9.

30. Tynkkynen J, Laatikainen T, Salomaa V, Havulinna AS, Blankenberg S, Zeller T, et al. NT-proBNP and the Risk of Dementia: A Prospective Cohort Study with 14 Years of Follow-Up. J Alzheimers Dis. 2014.

31. Kline R, Wong E, Haile M, Didehvar S, Farber S, Sacks A, et al. Peri-Operative Inflammatory Cytokines in Plasma of the Elderly Correlate in Prospective Study with Postoperative Changes in Cognitive Test Scores. Int J Anesthesiol Res. 2016;4(8):313-21.

32. Chen JM, Cui GH, Jiang GX, Xu RF, Tang HD, Wang G, et al. Cognitive impairment among elderly individuals in shanghai suburb, china: association of C-reactive protein and its interactions with other relevant factors. Am J Alzheimers Dis Other Demen. 2014;29(8):712-7.

33. O'Bryant SE, Johnson L, Edwards M, Soares H, Devous MD, Ross S, et al. The link between C-reactive protein and Alzheimer's disease among Mexican Americans. J Alzheimers Dis. 2013;34(3):701-6.

34. Komulainen P, Lakka TA, Kivipelto M, Hassinen M, Penttila IM, Helkala EL, et al. Serum high sensitivity C-reactive protein and cognitive function in elderly women. Age Ageing. 2007;36(4):443-8.

35. Alvarez A, Aleixandre M, Linares C, Masliah E, Moessler H. Apathy and APOE4 are associated with reduced BDNF levels in Alzheimer's disease. J Alzheimers Dis. 2014;42(4):1347-55.

36. Weinstein G, Seshadri S. Serum brain-derived neurotrophic factor as a predictor of incident dementia-reply. JAMA Neurol. 2014;71(5):653-4.

37. Aisen PS. Serum brain-derived neurotrophic factor and the risk for dementia. JAMA. 2014;311(16):1684-5.

38. Faria MC, Goncalves GS, Rocha NP, Moraes EN, Bicalho MA, Gualberto Cintra MT, et al. Increased plasma levels of BDNF and inflammatory markers in Alzheimer's disease. J Psychiatr Res. 2014;53:166-72.

39. Buerger K, Ernst A, Ewers M, Uspenskaya O, Omerovic M, Morgenthaler NG, et al. Blood-based microcirculation markers in Alzheimer's disease-diagnostic value of midregional pro-atrial natriuretic peptide/C-terminal endothelin-1 precursor fragment ratio. Biol Psychiatry. 2009;65(11):979-84.

40. Buerger K, Uspenskaya O, Hartmann O, Hansson O, Minthon L, Blennow K, et al. Prediction of Alzheimer's disease using midregional proadrenomedullin and midregional proatrial natriuretic peptide: a retrospective analysis of 134 patients with mild cognitive impairment. J Clin Psychiatry. 2011;72(4):556-63.

41. Schneider P, Buerger K, Teipel S, Uspenskaya O, Hartmann O, Hansson O, et al. Antihypertensive therapy is associated with reduced rate of conversion to Alzheimer's disease in midregional proatrial natriuretic peptide stratified subjects with mild cognitive impairment. Biol Psychiatry. 2011;70(2):145-51.

**Best plasma features for Point of Inception and Later Progression**

**Feature list dxb_12x27**

Alpha1MicroglobulinA1Microugml.bl

AngiotensinogenngmL.bl

Angiopoietin2ANG2ngmL.bl

ApolipoproteinAIIApoAIIngml.bl

ApolipoproteinAIVApoAIVugml.bl

ApolipoproteinDApoDugml.bl

ApolipoproteinEApoEugml.bl

BrainNatriureticPeptideBNPpgml.bl

ComplementC3C3mgmL.bl

CalcitoninpgmL.bl

CReactiveProteinCRPugmL.bl

Eotaxin3pgmL.bl

FasLigandFasLpgmL.bl

HeparinBindingEGFLikeGrowthFactorpgmL.bl

InsulinlikeGrowthFactorBindingProtengmL.bl

Interleukin16IL16pgmL.bl

Interleukin8IL8pgmL.bl

LeptinngmL.bl

MacrophageInflammatoryProtein1alphapgmL.bl

Vitronectinugml.bl

BetacellulinBTCpgmL.bl

HepatocyteGrowthFactorHGFngmL.bl

ImmunoglobulinAIgAmgmL.bl

MonokineInducedbyGammaInterferonMIpgml.bl

MatrixMetalloproteinase10MMP10ngml.bl

PancreaticPolypeptidePPPpgml.bl

PeptideYYPYYpgmL.bl

**Hot list**

AdiponectinugmL.bl

ApolipoproteinAIIApoAIIngml.bl

ApolipoproteinBApoBugml.bl

ApolipoproteinEApoEugml.bl

ApolipoproteinHApoHugmL.bl

Beta2MicroglobulinB2MugmL.bl

CD40LigandCD40LngmL.bl

ChromograninACgAngmL.bl

CReactiveProteinCRPugmL.bl

CystatinCngml.bl

MatrixMetalloproteinase7MMP7ngml.bl

InsulinuIUmL.bl

PlasminogenActivatorInhibitor1PAI1ngmL.bl

HaptoglobinmgmL.bl

Eotaxin1pgmL.bl

InterferongammaInducedProtein10IPpgml.bl

MacrophageColonyStimulatingFactor1ngmL.bl

MacrophageInflammatoryProtein1alphapgmL.bl

FollicleStimulatingHormoneFSHmIUmL.bl

Interleukin6receptorIL6rngmL.bl

SerumAmyloidPComponentSAPugmL.bl

SerotransferrinTransferrinmgdl.bl

TumorNecrosisFactoralphaTNFalphapgmL.bl

VitaminKDependentProteinSVKDPSugml.bl

LeptinngmL.bl
